# Hematology and biochemistry reference intervals for American crocodiles (*Crocodylus acutus*) in South Florida

**DOI:** 10.3389/fvets.2022.919488

**Published:** 2022-11-22

**Authors:** Sergio A. Balaguera-Reina, Nicole D. Jennings, Sidney T. Godfrey, Laura A. Brandt, Bryna Daykin, Michiko A. Squires, Frank J. Mazzotti

**Affiliations:** ^1^Department of Wildlife Ecology and Conservation, Fort Lauderdale Research and Education Center, University of Florida, Fort Lauderdale, FL, United States; ^2^United States Fish and Wildlife Service, Fort Lauderdale, FL, United States

**Keywords:** analytes, blood analysis, clinical diagnosis, conservation, wild populations

## Abstract

The American crocodile (*Crocodylus acutus*) is considered a vulnerable species by the International Union for Conservation of Nature (IUCN) Red List across its range and classified as locally threatened in several countries. There is a lack of knowledge involving hematological and physiological parameters in American crocodile populations, limiting our understanding of what are considered “normal” blood analyte results for the species and how to link them with health assessments. In this study, we analyzed 40 hematological and biochemical parameters and estimated reference intervals (RIs) for 35 of them based on 436 clinically healthy wild American crocodiles caught in South Florida between 2015 and 2021. Crocodiles were captured across three areas with different levels of human influence [low = Everglades National Park (ENP), medium = Biscayne Bay Estuary (BBE), and high = Turkey Point Nuclear Power Plant (TP)]. There was very strong-to-strong evidence for an effect of where animals were caught on five analytes: basophils %, phosphorus, proportion of (pr) alpha-2 globulins, absolute count (abs) of gamma globulins, and corticosterone, so no reference values were estimated but general statistics are presented and discussed. From the remaining analytes, we found no evidence that sex or size class had an effect on red blood cell (RBC), azurophils and monocytes abs, triglycerides, and albumin abs. However, we did find moderate-to-strong evidence that sex influenced azurophils % and size class influenced white blood cell (WBC), heterophils %, monocytes %, basophils abs, creatine phosphokinase (CPK), potassium, glucose, bile acids, alpha-1 globulin abs, and alpha-2 globulin pr and abs. Finally, there was strong evidence that both sex and size class influenced PCV, lymphocytes % and abs, eosinophils % and abs, aspartate aminotransferase (AST), calcium, sodium, chloride, total protein, albumin/globulin (A/G) ratio, albumin pr, alpha-1 globulin, and beta globulin abs. Intraspecific analysis showed that size is the variable that most influenced analytes explaining up to 29% of the variation, which relates to our findings based on intraindividual analysis. We compared our results with blood parameters reported for conspecifics as well as closely related species and discussed implication of those results for clinical diagnosis and American crocodile conservation.

## Introduction

The American crocodile (*Crocodylus acutus*) is one of 11 crocodylian species inhabiting the Americas, ranging from Florida in the United States to Venezuela across the Atlantic and Caribbean coasts and from Mexico to Peru on the Pacific coast (1). It is also one of three species (along with Cuban crocodile—*Crocodylus rhombifer*, and Orinoco crocodile—*C. intermedius*) currently cataloged under a threatened category by the International Union for Conservation of Nature (IUCN) Red List (2–4) across the continent. Studies regarding American crocodiles have been conducted through their range in many fields (e.g., ecology and genetics), yet a comprehensive set of hematological and plasma biochemical reference intervals (RIs) does not exist for either wild or captive populations. This knowledge gap limits our understanding of what could be considered within “normal” ranges for the species blood panel results and how to link them with health assessments. Dessauer (5) compiled the only four studies conducted on the topic for American crocodiles in the twentieth century (6–9) reporting average values for sodium, potassium, calcium, magnesium, chloride, bicarbonate, hemoglobin, and glucose. However, no data after the 1970s using current methods/equipment have been produced regarding hematology and plasma biochemistry in American crocodiles across its range.

Veterinary diagnostics in crocodylian have mainly relied on visual descriptive analysis and/or postmortem examination. However, visual analyses are limiting as it is known that these species do not exhibit early signs of discomfort, stress, or illness (10). More proactive/preventive methods such as blood testing are proven to be efficient to assess overall health conditions (11). Nonetheless, the current lack of baseline hematological and biochemical reference data or RI for many crocodylian species limits the application of this type of diagnostic tool to define criteria for health assessments (12, 13). Here, we assess 40 hematological and biochemical blood parameters (Supplementary Table 1) from clinically healthy (see methods) wild American crocodiles caught across South Florida and propose RIs for 35 analytes based on the American Society for Veterinary Clinical Pathology guidelines (14). We tested the effect of the area where animals were caught (high human influence vs. low-to-medium human influence) on analytes to avoid estimating RI from heterogeneous variables that can potentially mask habitat effects on hematological or biochemical parameters. Blood analyses performed in this study focused on factors that have been commonly used to assess overall health in reptiles in terms of nutrition, dehydration, and stress. We compared our findings with hematological and biochemical values reported for conspecifics as well as closely related species [Orinoco crocodile, freshwater crocodile (*C. johnstoni*), mugger crocodile (*C. palustris*), saltwater crocodile (*C. porosus*), Nile crocodile (*C. niloticus*), and Morelet's crocodile (*C. moreletii*)] and discussed the implication of those results for clinical diagnosis and American crocodile conservation.

## Materials and methods

### Ethical statements

This study was conducted at the University of Florida Institutional Animal Care and Use Committee (IACUC) protocol # 202109072, Department of the Interior National Park Service Scientific Research and Collection Permit EVER-SCI2020-0032 and BISC-SCI-2021-0002, and United States Fish and Wildlife Service threatened species permit # TE077258-5.

### Study area

**Figure 1:**
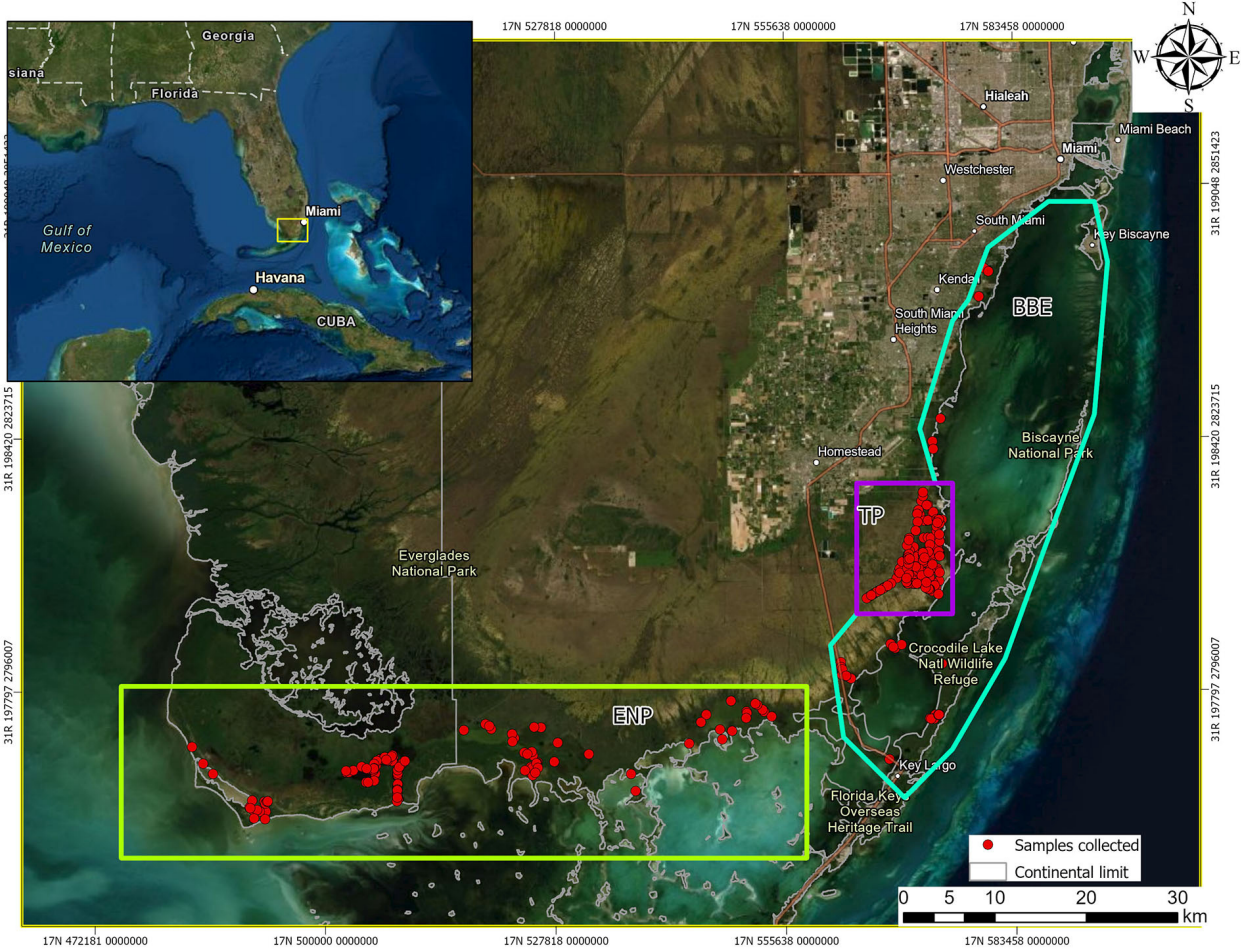
Geographic location of American crocodiles (*Crocodylus acutus*) captured and drawn blood samples from across South Florida, United States, between 2015 and 2021. Notice the three areas with different human influence level, Everglades National Park (ENP, low influence, green rectangle), Biscayne Bay Estuary (BBE, medium influence, blue–green polygon), and Turkey Point Power Plant (TP, high influence, purple square).

The study area is representative of available South Florida, United States habitats, and included three areas with varying levels of human influence: Everglades National Park (ENP; low), Biscayne Bay Estuary (BBE; medium), and Turkey Point Nuclear Power Plant (TP; high; Figure 1). ENP is a UNESCO International Biosphere Reserve and World Heritage Site, characterized by a very low relief with exposed and protected shorelines, creeks, ponds, small bays, coves, and a few man-made canals and ditches and low human interference (15). Shoreline habitats are commonly a mosaic of hardwood and buttonwood hammock and mangrove swamp (16). Interior protected habitats located landward of the exposed shoreline are commonly encompassed primarily by red and black mangrove (*Rhizophora mangle* and *Avicennia germinans*) with buttonwood (*Conocarpus erectus*) and hardwood hammocks habitats (17). BBE is a shallow, subtropical estuary that encompasses the shorelines of Biscayne Bay, Card Sound, and Barnes Sound (18). This area has been historically disturbed by human activities although its coastline remains largely undeveloped as most has been designated as protected (19). Red mangroves, black mangroves, white mangroves (*Laguncularia racemosa*), and buttonwoods dominate the exposed shoreline along the mainland through most of BBE (20) and mangroves as well as exotic species (Australian pine—*Casuarina spp*. and Brazilian pepper—*Schinus terebinthifolius*) dominate the protected shorelines of canals and ponds (18). Finally, TP is located in southeastern Miami-Dade County, Florida, and is owned by Florida Power and Light Co. This area is bordered by Biscayne National Park to the east and Card Sound to the south and is within federally designated critical crocodile habitat (21) serving as an important source of nesting for the American crocodile since 1978 when hatchlings were first captured (22). TP consists of a closed-loop series of 60-m-wide man-made cooling canals separated by 40-m-wide earthen berms that circulate water to cool the plant's condensers (21). Canal water temperature and salinity range from 34°C to 42°C in summer and 15ppt to 42ppt, respectively, depending on seasonal rains (23).

### Fieldwork and sampling

We captured wild American crocodiles of all size classes [hatchlings (TL < 65 cm), juvenile (65–150 cm), subadult (>150 <225 cm), and adult (≥225 cm)] (17) inhabiting coastal and marsh habitats of ENP, BBE, and TP at night between October 2015 through June 2021 using standard protocols and routes (24). We captured animals in late winter–early spring (January through March) and fall (October through December) and avoided sampling in summer (April through September) to circumvent unwanted effects due to reproductive behavior (mating and laying eggs) on blood parameters. Blood samples were collected as soon as possible after capture to minimize stress effects on blood values. The samples were withdrawn from the ventral coccygeal vein below the transverse process of tail vertebrate using either a 22G × 3/4-inch Luer-Lok tip with a 3-mL syringe or a 20 G × 1.5-inch Luer-Lok tip with a 6-mL syringe (BD Medical Company, New Jersey, USA) depending on the size of the animal (the former for hatchlings and the latter for other size classes). The volume of collected blood was limited by the weight of the individual, not to exceed 1% of the total weight. Depending on the amount of blood collected, the samples were transferred into either a 3-mL BD lithium heparin vacutainer (BD Medical Company, Franklin Lakes, NJ, USA) or microvette 500-μL lithium heparin vials (Sarstedt Inc., Nümbrecht, Germany) and inverted seven to eight times to ensure even mixing. Blood smears were not performed at the time of blood draw due to humid and rainy field conditions. Instead, the vials were stored in a cooler with an insulating layer to avoid direct contact of samples with ice to minimize a risk of freezing and lysing blood cells as recommended by Finger et al. (25).

Total length (TL), snout–vent length, weight, sex, capture location (Universal Transverse Mercator, WGS84), capture time, and time of blood draw were also recorded. To ensure representation of healthy crocodiles from the South Florida population, we excluded sampling from individuals that presented major deformities and recognizable health issues (e.g., emaciated, with open wounds, or visually lacerated) due to possible effects it could have on evaluated parameters. All American crocodiles were released at their capture site after sample and data collection.

### Hematology and plasma biochemical analysis

We transferred the blood samples within 12 h of collection to the University of Miami's Comparative Pathology Laboratory, Florida, USA, for analysis. Blood smears were prepared on full slides. Complete blood counts (CBC) with differential were performed using the Natt and Herrick method (26) with Wright–Giemsa stain at 1,000X (microscopic magnification). One hundred cells were counted, which is a standard procedure for non-mammalian vertebrate. Natt and Herrick's stain (Vetlab, Miami, Florida, USA) and a hemacytometer with the improved Neubauer counting chamber (VWR International, Radnor, Pennsylvania, USA) were used for absolute (abs) white and red blood cell counts. Packed cell volume (PCV) was determined using a Haematokrit 200 centrifuge (Andreas Hettich GimbH & Co. KG, Tuttlingen, Germany) at 10,000 rpm (9,903 g) for 5 min. Blood plasma was obtained by spinning samples for 5 min at 10,000 rpm (9,391 g) in an Eppendorf 5,254 centrifuge (Eppendorf AG, Hamburg, Germany). Plasma biochemistry was performed using Vitros 250 (Ortho, Rochester, New York, USA), bile acids, and hydroxybutyrate reagents (Randox, Kearneysville, West Virginia, USA) with RX Daytona (Randox). Finally, total protein, albumin/globulin (A/G) ratio, albumin, alpha-1 globulins, alpha-2 globulins, beta globulins, and gamma globulins were measured *via* electrophoresis using Helena reagents and a SPIFE 300 analyzer (Helena, Beaumont, Texas, USA). A comprehensive list with all the analytes measured in American crocodiles in the present study is given in Supplementary Table 1. All analyses described above were performed within 24 h of sample collection. The plasma was banked at −80°C for later corticosterone analysis, which was completed in batches at the end of the sampling season. The samples were rejected if clotted, diluted, suspected to contain lymph, or with high hemolysis (≥2) and lipemia (≥3) index values due to the effect these variables had on chemical plasma composition (27). Finally, biochemical values reported as outside the instruments range (above or below) were excluded from analysis due to the uncertainty of the actual value (e.g., >1,600).

### Data analysis

Data were screened for crocodiles captured more than once (recaptures), and only data for the most recent capture were used for RIs calculations to avoid violations of basic statistical assumptions (independence of the data). Data from recaptured crocodiles were used for intraindividual analysis to understand changes through time across analytes. All analytes were assessed for normality (Shapiro–Wilk test) and homoscedasticity (Fligner–Killeen test) to define the most appropriate statistical approach using R (28). We defined statistical evidence as very strong (*p*-value ≤ 0.001), strong (*p*-value ≤ 0.01), moderate (*p*-value ≤ 0.05), weak (*p*-value ≤ 0.10), or little-to-no evidence (*p*-value > 0.10) as suggested by Bland (29) and Muff (30). Analytes coming from samples collected in high human-influenced areas (TP) were tested for equality of means (Wilcoxon's rank sum test) against samples collected in medium-to-low influenced areas (ENP and BBE) to ensure that environmental conditions at the capture site did not affect the hematological and biochemical composition. Once analytes not strongly affected by area (very strong-to-strong evidence, see results) were identified, we performed the analysis of variance (Kruskal–Wallis tests and Dunn's pairwise test) with Bonferroni correction as a whole and by age group and sex to reveal any effect caused by these two variables. Individuals unsuccessfully sexed were excluded from analysis when grouped by sex to gain a clear understanding of the effect of this variable across analytes.

Finally, we estimated 95 % RIs and associated 90% confidence intervals (CI) using non-parametric (*n* ≥ 120) and robust Box–Cox transformed (*n* ≥ 20) analyses as defined by the American Society for Veterinary Clinical Pathology guidelines (14) *via* Reference Value Advisor Excel macroinstructions (31). Friedrichs et al. (14) recommend 40 as the minimum number of samples to be used to estimate 95% non-parametric RIs so analytes with sample size between 20 and 40 were given special attention. In cases where the robust Box–Cox transformation did not describe the data as expected (distribution was not Gaussian) (31), we presented only descriptive statistics (mean, standard deviation (SD), median, max, and min). However, on those cases, we presented overall RIs that can be used as reference for the species. It is important to notice that these overall RIs are likely affected by size class and/or sex (see results) so it should be used with caution. Data were tested for outliers using the Tukey and Dixon methods as recommended by the reference value advisor software (31). Nonetheless, as recommended by Friedrichs et al. (14), unless outliers were known to be aberrant observations, our emphasis was on retaining rather than on deleting them. Based on statistical findings (see results), we estimated general reference intervals for 35 out of the 40 analytes assessed on American crocodiles across South Florida, five including all individuals (no effect), one grouped by sex, 12 grouped by size classes, and 17 grouped by sex and size class. We assessed relationships between analytes and the covariates affecting them (analyte ~ TL, analyte ~ TL + sex) *via* linear regression models using the *lm* function in R. Categorical data (sex) were translated to dummy variables (0 or 1 indicating the absence or presence of categorical effect, respectively) using the “fastDummies” package.

## Results

We collected and processed a total of 507 blood samples from 436 American crocodiles (193 hatchlings, 167 juveniles, 55 subadults, and 21 adults; 136 females, 184 males, and 116 unknown) across the study area. Of those individuals, we recaptured 61 animals (mean days between first and last capture 325.1 ± 291.9) resampling blood two times from 54 individuals (294.4 ± 290.4 days between first and second capture), three times from four individuals (663 ± 166.7 days between first and third capture), and four times from three individuals (427 ± 96.3 days between first and fourth capture). We excluded 14 samples from analyses due to hemolysis and lipemia index values equal to or greater than 2 and 3, respectively, as recommended by Stacy (27). Total length and weight of individuals overall ranged from 34.8 cm to 309.5 cm and from 53 gr to 88,000 gr and had a mean and standard deviation (SD) of 77.88 ± 57.52 cm and 1,263.9 ± 12,578 g, respectively.

Lymphocytes (59 ± 19 %) were the most frequent leukocyte, followed by heterophils (24 ± 19 %), eosinophils (9 ± 9 %), basophils (5 ± 4 %), monocytes (4 ± 4 %), and azurophils (0.5 ± 1.9 %). Analytes did not fit assumption of samples coming from a normally distributed population except for proportion of (pr) albumin (Shapiro–Wilk *p*-value = 0.24). However, we found very strong-to-moderate evidence of homogeneity of variance across 20 and 21 out the 40 analytes when assessed by sex and size class, respectively (Supplementary Table 1). We found very strong-to-strong evidence that the area where animals were caught (high human-influenced TP N = 164 vs. low-to-medium human-influenced ENP and BBE N = 272) has an effect on five out of the 40 analytes assessed (basophils %, phosphorus, alpha-2 globulin abs, gamma globulin abs, and corticosterone) and moderate evidence of the same effect on six analytes (heterophils abs, aspartate aminotransferase—AST, chloride, beta globulin pr and abs, and pr gamma globulin; Table 1). These analytes were higher in TP compared with ENP and BBE except for phosphorous, AST, and pr beta globulin.

**Table 1:** General statistics of American crocodile (*Crocodylus acutus*) hematological and biochemical analytes with very strong-to-moderate evidence of being affected by areas (high human influenced—Turkey Point Nuclear Power Plant vs. low-to-medium human influenced—Everglades National Park—ENP and Biscayne Bay Estuary—BBE) in South Florida, United States, based on an equal means test (Wilcoxon's sum rank test).

**Analytes**	**Turkey Point (*****N*** = **164)**		**ENP and BBE (*****N*** = **272)**		***p*-value**
	**Mean (SD)**	**Median [Min, Max]**	**Mean (SD)**	**Median [Min, Max]**	
Basophils %	5.74 (4.47)	5 [0.0, 21]	4.61 (4.40)	4 [0.0, 19.0]	0.004
Phosphorus mg/dL	5.40 (1.46)	5.20 [1.60, 10.8]	5.91 (1.55)	5.80 [2.50, 12.3]	0.001
Alpha-2 globulins g/L	10.4 (5.65)	10.6 [0.56, 28.6]	8.95 (8.83)	9.20 [0.53, 122]	0.003
Gamma globulins g/L	4.24 (2.36)	4.30 [0.21, 12.2]	3.63 (3.63)	3.20 [0.17, 43.1]	0.000
Corticosterone nmol/L	53.1 (49.4)	36.6 [2.76, 272]	36.1 (37.3)	24.2 [0.83, 232]	0.000
Heterophils abs. 10^9^/L	2.59 (1.97)	2.11 [0.16, 10.8]	2.66 (2.99)	1.68 [0, 20.6]	0.044
AST ukat/L	1.03 (0.651)	0.89 [0.20, 4.36]	1.16 (0.769)	0.94 [0.37, 6.13]	0.030
Chloride mmol/L	121 (9.63)	121 [91.0, 151]	120 (8.59)	119 [98.0, 148]	0.046
Proportion of beta globulins	0.42 (0.05)	0.41 [0.25, 0.55]	0.43 (0.04)	0.43 [0.31, 0.63]	0.031
Beta globulins g/L	17.4 (8.68)	18.4 [0.77, 37.1]	16.1 (18.4)	16.7 [1.02, 274]	0.047
Proportion of gamma globulins	0.10 (0.03)	0.10 [0.04, 0.23]	0.10 (0.03)	0.09 [0.04, 0.21]	0.025

From these analytes, we decided not to estimate reference intervals for those with very strong-to-strong evidence (p-value ≤ 0.01) to ensure that environmental conditions at the capture site did not influence the hematological and biochemical composition reported. Analytes with moderate evidence were used to calculate RIs and are given in Tables 2, 3.

When evaluating the 35 analytes with moderate-to-no evidence of difference in means across South Florida, we found no evidence that either sex or size class has an effect on red blood cells (RBC), azurophils and monocytes abs, triglycerides, and albumin abs (Table 2). However, we did find strong evidence that sex has an effect on azurophils %, moderate-to-very strong evidence that size class has an effect on white blood cells (WBC), heterophils %, monocytes %, heterophils abs, creatine phosphokinase (CPK), potassium, glucose, uric acid, pr alpha-2 globulin, beta globulins pr and abs, and bile acids (Table 3), and moderate-to-very strong evidence that both sex and size class have an effect on PCV, lymphocytes % and abs, eosinophils % and abs, basophils abs, AST, calcium, sodium, chloride, total protein, albumin/globulin (A/G) ratio, pr albumin, alpha-1 globulin pr and abs, pr gamma globulin, and hydroxybutyrate (Table 4).

**Table 2:** American crocodile (*Crocodylus acutus*) hematological and biochemical general statistics and reference values with 90% confidence intervals (CI) for wild populations in South Florida, United States.

**Blood Parameter**	***n***	**Mean**	**Median**	**Category**	***p*-value**	**Reference**	**CI lower**	**CI upper**
		**(SD)**	**[Min, Max]**		**SC—Sex**	**interval**	**limit**	**limit**
Red blood cell count 10^9^/L	380	0.76 (0.30)	0.75 [0.10, 1.87]	CBC	0.11–0.59	0.18–1.45	0.14–0.25	1.30–1.59
Azurophil abs 10^9^/L	373	0.01 (0.05)	0 [0, 0.47]	CBC	0.75–0.10	0.00–0.07	0.00–0.00	0.00–0.36
Monocyte abs 10^9^/L	375	0.424 (0.48)	0.28 [0, 2.63]	CBC	0.42–0.49	0.00–1.78	0.00–0.00	1.53–2.15
Triglycerides mmol/L	430	1.13 (1.22)	0.66 [0.06, 6.54]	CHE	0.46–0.55	0.10–4.41	0.10–0.10	4.00–4.85
Albumin g/L	429	9.82 (3.35)	9.91 [0.26, 20]	PE	0.26–0.12	1.13–16.13	0.82–2.70	15.60–18.16

Notice that we found no evidence that these analytes were significantly affected by either size class or sex (p-value). Aberrant values were excluded from analysis as recommended by Friedrichs et al. (14) and are reported at the bottom of the table for reference. Abs, Absolute counts; SD, Standard Deviation; CBC, complete blood count; CHE, Chemistry; PE, Protein Electrophoresis.

Aberrant values found in red blood cells counts = 10.9 10^9^/L; Azurophil absolute counts = 0.78, 1.14, and 2.04 10^9^/L; Monocytes absolute counts = 5.47 10^9^/L; Triglycerides = 11.59, 12.69, 13.18, and 16.03 mmol/L; Albumin = 117.6 g/L.

**Table 3:** Hematological and biochemical general statistics and reference values with 90% confidence intervals (CI) for wild American crocodile (*Crocodylus acutus*) analytes that showed statistical evidence of being affected either by sex or by size class in South Florida, United States.

**Blood parameter**	**Group**	***N***	**Mean**	**Median**	**Cat**	***p*-value**	**Reference**	**CI lower**	**CI upper**
			**(SD)**	**[Min, Max]**			**interval**	**limit**	**limit**
Azurophil %	F	133	0.0 (1)	0.0 [0.0, 7]	CBC	0.005	0.0–5	0.0–0.0	4–7
	M	177	0.0 (1)	0.0 [0.0, 8]			0.0–4	0.0–0.0	0.0–8
White blood cell count 10^9^/L	Hatchling	178	11.6 (4.63)	11.1 [2.00, 32.4]	CBC	0.000	4.63–23.91	2.00–5.30	18.30–32.40
	Juvenile	160	13.7 (4.51)	13.0 [3.50, 26.3]			5.70–23.00	3.50–7.30	21.30–26.30
	Subadult	54	13.1 (4.99)	12.9 [5.00, 33.7]			5.38–30.10	5.00–7.70	21.93–33.70
	Adult	21	10.9 (4.26)	10.0 [5.00, 22.2]			4.18–22.18	3.37–5.59	17.83–26.98
Heterophils %	Hatchling	184	29 (20)	24 [1, 86]	CBC	0.000	5–78	1–6	74–86
	Juvenile	163	20 (18)	13 [1, 88]			2–79	1–3	64–88
	Subadult	53	18 (14)	14 [2, 62]			2–60	2–4	49–62
	Adult	21	27 (21)	20 [6, 87]			5–86	ND−6	54–100
Monocytes %	Hatchling	157	4 (4)	3 [0, 18]	CBC	0.021	0.0–13	0.0–0.0	11–18
	Juvenile	149	3 (3)	2 [0, 15]			0.0–14	0.0–0.0	11–15
	Subadult	49	3 (3)	2 [0, 13]			0.0–12	0.0–0.0	10–13
	Adult	19	5 (3.7)	5 [0, 12]			NA	NA	NA
	Overall	376	3.6 (3.8)	2.0 [0, 23]			0.0–13.6	0.0–0.0	12.0–14.6
Heterophil abs 10^9^/L	Hatchling	156	2.72 (2)	2.25 [0, 10.79]	CBC	0.016	0.39–9.25	0.00–0.50	7.05–10.79
	Juvenile	148	2.35 (2.37)	1.57 [0, 12]			0.20–10.49	0.00–0.36	7.92–12
	Subadult	47	1.81 (1.17)	1.64 [0.13, 5.2]			0.16–5.18	0.13–0.38	3.43–5.2
	Adult	18	2.09 (1.49)	1.62 [0.40, 6.24]			NA	NA	NA
	Overall	376	2.63 (2.66)	1.86 [0.0, 20.6]			0.27–10.24	0.16–0.39	9.18–11.29
CPK ukat/L	Hatchling	187	115.70 (109.14)	81.18 [2.57, 460.20]	CHE	0.000	3.23–387.59	2.57–6.15	340.06–460.20
	Juvenile	162	68 (79.51)	28.8 [2.09, 380.43]			2.96–327.59	2.09–4.69	259.52–380.43
	Subadult	52	26.82 (31.05)	13.35 [2.47, 133.55]			2.71–128.81	2.47–5.72	98.61–133.55
	Adult	17	16.59 (19.06)	12.91 [0.75, 82.80]			NA	NA	NA
	Overall	427	90.01 (115.5)	32.43 [0.75, 917.1]			3.06–382.30	2.54–3.73	340.33–434.2
Potassium mmol/L	Hatchling	193	4.87 (0.83)	4.80 [3.20, 7.40]	CHE	0.024	3.40–7.03	3.30–3.60	6.30–7.40
	Juvenile	167	4.85 (1.08)	4.70 [2.90, 8.80]			3.32–7.28	2.90–3.50	6.80–8.80
	Subadult	55	5.18 (1.08)	5.30 [3.30, 7.30]			3.34–7.14	3.30–3.64	6.70–7.30
	Adult	21	5.38 (1.29)	5.50 [2.80, 7.90]			2.58–8.12	1.82–3.55	7.31–8.90
Glucose mmol/L	Hatchling	193	4.07 (0.88)	4.05 [2.05, 6.22]	CHE	0.000	2.49–5.96	2.16–2.78	5.55–6.16
	Juvenile	167	3.63 (1.04)	3.50 [1.72, 6.94]			2.18–5.94	1.72–2.28	5.61–6.94
	Subadult	55	3.68 (1.04)	3.61 [1.55, 5.61]			1.71–5.52	1.55–2.12	5.22–5.61
	Adult	21	4.06 (1.08)	4.00 [1.55, 6.83]			1.84–6.40	1.21–2.67	5.49−7.23
	Overall	436	3.85 (0.99)	3.77 [1.55, 6.94]			2.16–5.94	2.05–2.27	5.61–6.06
Uric acid mg/dL	Hatchling	192	8.06 (3.92)	7.20 [2.00, 22.4]	CHE	0.000	2.28–16.50	2.20–2.70	15.50–22.40
	Juvenile	167	7.23 (3.88)	6.30 [1.40, 22.4]			1.74–16.68	1.40–2.60	14.10–22.40
	Subadult	55	6.32 (4.60)	5.20 [1.00, 24.7]			1.04–21.62	1.00–1.38	14.10–24.70
	Adult	21	5.30 (3.58)	5.60 [0.50, 13.3]			NA	NA	NA
	Overall	436	7.51 (4.39)	6.50 [0.50, 36.50]			1.59–16.91	1.39–1.90	15.51–22.03
Bile acids μmol/L	Hatchling	185	14 (10.1)	12.1 [0.80, 53.6]	CHE	0.000	1.07–43.46	0.80–2.10	35.3–53.6
	Juvenile	161	8.63 (7.38)	6.20 [0.10, 36.3]			1.00–28.38	0.10–1	24.60–36.3
	Subadult	53	5.99 (5.51)	4.90 [0, 23.5]			0.04–23.19	0.00–0.38	15.40–23.50
	Adult	19	7.01 (6.26)	4.50 [1.00, 18.9]			NA	NA	NA
	Overall	424	11.47 (11.62)	8.30 [0.0, 101.9]			0.90–43.65	0.69–1.0	35.67–53.60
Proportion of alpha-2 globulins	Hatchling	192	0.25 (0.03)	0.25 [0.17, 0.34]	PE	0.001	0.18–0.33	0.18–0.19	0.31–0.34
	Juvenile	164	0.24 (0.04)	0.24 [0.14, 0.41]			0.18–0.31	0.14–0.19	0.30–0.41
	Subadult	54	0.24 (0.04)	0.23 [0.16, 0.32]			0.17–0.31	0.16–0.19	0.30–0.32
	Adult	20	0.23 (0.04)	0.23 [0.17, 0.35]			NA	NA	NA
	Overall	430	0.25 (0.04)	0.24 [0.14, 0.41]			0.18–0.32	0.17–0.18	0.31–0.33
Proportion of beta globulins	Hatchling	192	0.41 (0.05)	0.41 [0.31, 0.63]	PE	0.000	0.32–0.51	0.31–0.34	0.48–0.57
	Juvenile	164	0.43 (0.05)	0.43 [0.25, 0.55]			0.35–0.53	0.25–0.36	0.50–0.55
	Subadult	54	0.45 (0.04)	0.45 [0.37, 0.54]			0.37–0.53	0.37–0.39	0.50–0.54
	Adult	20	0.45 (0.04)	0.44 [0.38, 0.55]			0.38–0.58	0.37–0.40	0.51–0.69
Beta Globulins g/L	Hatchling	192	14.5 (8.37)	15.7 [0.77, 39.1]	PE	0.000	1.15–31.65	0.86–1.26	26.80–34.70
	Juvenile	163	16.83 (8.78)	19.2 [1.21, 37.1]			1.51–31.21	1.21–1.85	28.50–37.10
	Subadult	54	16.2 (11.3)	19.7 [1.29, 35.9]			1.35–35.56	1.29–1.86	32.26–35.90
	Adult	20	23.2 (12.8)	24.6 [1.75, 49.0]			NA	NA	NA
	Overall	430	16.60 (15.57)	17.75 [0.77, 274.4]			1.26–33.61	1.16–1.36	31.74–37.0

Size class was determined by total length (hatchling ≤ 65 cm, juvenile 65 ≤ 150 cm, subadult 151 ≤ 225 cm, and adult ≥ 225 cm) as recommended by Mazzotti (17). Reference intervals for hatchlings, juveniles, and subadults were estimated based on non-parametric analysis and for adults were estimated using robust Box–Cox transformed analysis due to insufficient samples to run non-parametric reference intervals. In the cases when we had an insufficient number of samples to obtain reference or confidence intervals (NA), we estimated an overall (including all data) RIs. It is important to notice that these overall RIs are likely affected by size class (see results) so it should be used with caution. Aberrant values were excluded from analysis as recommended by Friedrichs et al. (14) and are reported at the bottom of the table for reference. M, male; F, female; N, sample size; SD, standard deviation; abs, Absolute count; Cat, Category; CBC, complete blood count; CHEM, Chemistry; PE, Protein electrophoresis; Cat, Category.

Aberrant values found in Azurophils males =16 %, females = 18 %; Heterophils subadults = 85 %; Monocytes subadults = 21 and 23 %; Heterophils absolute counts hatchlings = 20.64 10^9^/L, juveniles 18.17 10^9^/L, subadults = 8, 9.54, 9.92, and 11.19 10^9^/L, and adults = 19.31 10^9^/L; CPK hatchlings = 662.70 and 837.35 ukat/L, juveniles = 500.48 and 917.06 ukat/L, subadults = 164.95 and 239.64 ukat/L, and adults = 145.99, 221.07, and 416.11 ukat/L; Uric acid hatchlings = 29.3 and 36.5 mg/dL; Bile acid hatchlings = 68.8, 74, and 76.5 μmol/L, juveniles = 49.2, 54.4, and 101.9 μmol/L; Beta globulins juveniles = 274.4 g/L.

**Table 4:** Hematological and biochemical general statistics and reference values with 90% confidence intervals (CI) for wild American crocodile (*Crocodylus acutus*) analytes that showed statistical evidence of being affected by both sex and size class in South Florida, United States.

**Blood parameter**	**Group**	***N***	**Mean (SD)**	**Median [Min, Max]**	**Cat**	***p*-value sex—SC**	**Reference interval**	**CI lower limit**	**CI upper limit**
PCV	F—Hatchling	21	18.9 (4.32)	19 [10, 27]	CBC	0.025–0.000	9–28	7–13	25–31
	F—Juvenile	58	20.7 (5.16)	21 [5, 38]			9–36	5–14	28–38
	F—Subadult	40	21.5 (4.59)	21.5 [12, 36]			12–36	12–15	27–36
	F—Adult	11	20.5 (4.18)	20 [15, 29]			NA	NA	NA
	M—Hatchling	68	18.9 (4.07)	18 [12, 30]			12–29	12–13	27–30
	M—Juvenile	78	20.2 (5.03)	20 [9, 37]			11–34	9–14	29–37
	M—Subadult	14	19.4 (4.88)	20 [11, 27]			NA	NA	NA
	M—Adult	9	20.1 (3.48)	19 [17, 29]			NA	NA	NA
	Overall	407	19 (5.0)	19 [5, 38]			9.2–29	8.2–11	29–32
Lymphocytes %	F—Hatchling	21	45.5 (18.1)	53 [15, 69]	CBC	0.000–0.000	0.0–82	0.0–21	73–89
	F—Juvenile	61	61.1 (17.3)	65 [19, 93]			21–92	19–28	85–93
	F—Subadult	40	53.3 (19.7)	54.5 [11, 87]			11–87	11–29	79–87
	F—Adult	12	39.6 (20.6)	34 [6, 79]			NA	NA	NA
	M—Hatchling	71	65.2 (17.4)	70 [15, 90]			19–89	15–23	85–90
	M—Juvenile	84	65.2 (16.7)	67.5 [9, 94]			23–90	9–33	86–94
	M—Subadult	14	50.7 (21.9)	50.5 [8, 77]			NA	NA	NA
	M—Adult	9	47.1 (15.4)	48 [30, 77]			NA	NA	NA
	Overall	422	58.7 (19.4)	62 [6, 96]			14.2–88	11–18.6	86–90
Eosinophils %	F—Hatchling	21	6.57 (3.63)	6 [0, 13]	CBC	0.000–0.000	0.0–15	0.0–1	12–17
	F—Juvenile	61	10.4 (7.82)	10 [0, 33]			0.0–31	0.0–1	27–33
	F—Subadult	40	17.7 (12.1)	13 [1, 51]			1–51	1–5	39–51
	F—Adult	12	21.3 (18.8)	16 [3, 64]			NA	NA	NA
	M—Hatchling	71	3.48 (3.14)	3 [0, 14]			0.0–51	1–5	39–51
	M—Juvenile	84	8.68 (7.33)	7.5 [0, 33]			0.0–33	0.0–1	25–33
	M—Subadult	14	14.9 (7.95)	15 [1, 29]			NA	NA	NA
	M—Adult	9	15.3 (13.6)	11 [2, 38]			NA	NA	NA
	Overall	421	8.5 (9)	6.0 (0–64)			0–36	0.0–0.0	33–38
Lymphocyte abs	F—Hatchling	15	6.06 (2.49)	6.15 [1.36, 10.68]	CBC	0.015–0.000	NA	NA	NA
10^9^/L	F—Juvenile	55	8.21 (3.94)	8.24 [0, 17.9]			0.32–17.54	0.00–2.29	14.92–17.89
	F—Subadult	37	6.72 (4.59)	6.25 [1.60, 17.83]			1.65–15.4	1.35–2.30	12.78–18.36
	F—Adult	11	3.84 (1.78)	3.92 [1.33, 7.90]			NA	NA	NA
	M—Hatchling	69	7.50 (3.14)	7.63 [1.00, 16.6]			1.38–14.50	1.00–2.88	12.38–16.58
	M—Juvenile	81	9.23 (3.93)	8.82 [1.35, 21.3]			2.42–20.00	1.35–3.99	15.78–21.25
	M—Subadult	13	6.34 (3.11)	6.90 [0.66, 11.9]			NA	NA	NA
	M—Adult	8	5.21 (2.95)	5.32 [1.90, 9.39]			NA	NA	NA
	Overall	376	7.4 (3.81)	7.02 [0.0–22.92]			1.35–16.83	0.99–1.70	15.05–18.58
Eosinophil abs	F—Hatchling	16	0.959 (0.501)	0.99 [0.28, 1.96]	CBC	0.000–0.000	NA	NA	NA
10^9^/L	F—Juvenile	54	1.28 (1.06)	1.06 [0, 5.17]			0.00–4.66	0.00–0.07	3.20–5.17
	F—Subadult	37	2.21 (1.43)	1.86 [0.53, 6.60]			0.44–6.28	0.32–0.63	4.92–8
	F—Adult	10	1.78 (1.22)	1.57 [0.15, 3.53]			NA	NA	NA
	M—Hatchling	69	0.420 (0.431)	0.22 [0, 2.13]			0.00–1.79	0.00–0.00	1.27–2.13
	M—Juvenile	79	1.1 (0.9)	0.83 [0, 3.95]			0.00–3.62	0.00–0.14	2.56–3.95
	M—Subadult	13	1.97 (1.29)	1.70 [0, 4.64]			NA	NA	NA
	M—Adult	8	1.52 (1.05)	1.40 [0.19, 3.36]			NA	NA	NA
	Overall	374	1.06 (1.15)	0.67 [0.0–7.03]			0.0–4.47	0.0–0.0	3.56–5.55
Basophil abs 10^9^/L	F—Hatchling	16	0.636 (0.619)	0.535 [0, 2.13]	CBC	0.033–0.000	NA	NA	NA
	F—Juvenile	55	0.867 (0.789)	0.53 [0, 2.76]			0.00–2.64	0.00–0.00	2.26–2.76
	F—Subadult	36	0.85 (0.52)	0.79 [0, 2.41]			NA	NA	NA
	F—Adult	11	0.524 (0.432)	0.65 [0, 1.28]			NA	NA	NA
	M—Hatchling	68	0.43 (0.39)	0.28 [0, 1.56]			0.00–1.49	0.00–0.00	1.13–1.56
	M—Juvenile	81	0.751 (0.633)	0.63 [0, 2.38]			0.00–2.36	0.00–0.00	2.23–2.38
	M—Subadult	13	0.705 (0.637)	0.58 [0, 1.84]			NA	NA	NA
	M—Adult	8	0.723 (0.542)	0.665 [0.16, 1.74]			NA	NA	NA
	Overall	374	0.64 (0.61)	0.49 [0.0–2.91]			0.0–2.26	0.0–0.0	2.12–2.38
AST ukat/L	F—Hatchling	21	1.25 (0.43)	1.22 [0.53, 2.22]	CHE	0.002–0.000	0.488–2.27	0.36–0.67	1.91–2.67
	F—Juvenile	59	0.74 (0.28)	0.67 [0.32, 1.55]			0.33–1.53	0.32–0.40	1.25–1.55
	F—Subadult	38	0.70 (0.23)	0.68 [0.37, 1.55]			NA	NA	NA
	F—Adult	12	0.650 (0.319)	0.593 [0.200, 1.37]			NA	NA	NA
	M—Hatchling	71	1.22 (0.53)	1.07 [0.43, 3.47]			0.49–2.50	0.43–0.63	2.08–3.47
	M—Juvenile	85	0.83 (0.33)	0.73 [0.37, 2.07]			0.38–1.73	0.37–0.42	1.50–2.07
	M—Subadult	15	0.66 (0.255)	0.601 [0.317, 1.10]			NA	NA	NA
	M—Adult	8	0.735 (0.203)	0.693 [0.484, 1.05]			NA	NA	NA
	Overall	428	1.11 (0.73)	0.92 [0.20, 6.13]			0.38–3.0	0.37–0.42	2.63–3.82
Calcium mmol/L	F—Hatchling	22	2.99 (0.222)	3.00 [2.53, 3.38]	CHE	0.004–0.000	2.45–3.41	2.28–2.64	3.30–3.51
	F—Juvenile	60	3.08 (0.290)	3.09 [2.45, 3.83]			2.49–3.79	2.45–2.62	3.55–3.83
	F—Subadult	38	3.17 (0.26)	3.19 [2.63, 3.83]			2.66–3.72	2.54–2.79	3.58–3.85
	F—Adult	12	4.17 (1.47)	3.58 [2.13, 7.43]			NA	NA	NA
	M—Hatchling	72	2.99 (0.221)	2.98 [2.50, 3.60]			2.52–3.50	2.50–2.66	3.38–3.60
	M—Juvenile	86	3.04 (0.306)	3.04 [2.28, 3.95]			2.33–3.87	2.28–2.68	3.58–3.95
	M—Subadult	15	3.10 (0.404)	3.18 [2.25, 3.78]			NA	NA	NA
	M—Adult	8	3.27 (0.275)	3.36 [2.83, 3.60]			NA	NA	NA
	Overall	423	3.02 (0.30)	3.0 [2.13, 4.0]			2.45–3.69	2.32–2.53	3.56–3.80
Sodium mmol/L	F—Hatchling	22	148 (9.14)	147 [133, 161]	CHE	0.000–0.000	127.92–167.76	124.03–134.23	162.17–172.73
	F—Juvenile	62	152 (10.5)	151 [134, 182]			135.15–176.83	134.00–137.73	170.30–182.00
	F—Subadult	40	158 (9.65)	157 [138, 180]			138.10–179.90	138.00–145.00	173.98–180.00
	F—Adult	12	162 (7.14)	166 [147, 171]			NA	NA	NA
	M—Hatchling	74	145 (6.27)	145 [130, 166]			132.63–161.63	130.00–136.75	155.63–166.00
	M—Juvenile	86	149 (9.49)	147 [131, 184]			133.53–178.73	131.00–137.35	163.65–184.00
	M—Subadult	15	157 (12.9)	162 [132, 175]			NA	NA	NA
	M—Adult	9	159 (9.99)	164 [145, 171]			NA	NA	NA
	Overall	436	149.9 (10)	148 [130, 184]			134.9–172	133–136	171–174.2
Chloride mmol/L	F—Hatchling	22	122 (9.90)	122 [101, 135]	CHE	0.005–0.000	96.7–139.9	88.2–104.7	134.9–143.5
	F—Juvenile	62	122 (7.63)	122 [108, 141]			108.00–139.85	108.00–109.15	133.00–141.00
	F—Subadult	40	124 (8.45)	125 [103, 139]			103.08–138.98	103.00–110.08	135.00–139.00
	F—Adult	12	123 (5.53)	124 [115, 135]			NA	NA	NA
	M—Hatchling	74	121 (7.93)	120 [103, 151]			107.38–141.38	103.00–110.88	135.00–151.00
	M—Juvenile	86	118 (10.0)	118 [91.0, 148]			96.30–138.55	91.00–103.45	135.04–148.00
	M—Subadult	15	123 (7.10)	123 [113, 137]			NA	NA	NA
	M—Adult	9	124 (7.18)	122 [114, 135]			NA	NA	NA
	Overall	435	120.2 (9)	120 [91, 151]			103–139.1	101–106	137.1–141
Total Protein g/L	F—Hatchling	22	47.0 (9.97)	49.0 [28.0, 70.0]	CHE	0.000	26.84–70.28	20.94–33.90	62.73–75.16
	F—Juvenile	60	50.7 (10.4)	52.0 [24.0, 78]			26.1–74.9	24–32	67–78
	F—Subadult	39	53.5 (11.7)	54.0 [30.0, 82.0]			30.68–78.83	25.71–36.85	72.53–85.09
	F—Adult	12	63.4 (24.7)	68.0 [11.0, 110]			NA	NA	NA
	M—Hatchling	74	45.3 (12.5)	43.0 [22.0, 90.0]			22.00–72.50	22.00–27.50	70.00–90.00
	M—Juvenile	86	47.5 (12.0)	48.0 [12.0, 72.0]			22.35–71.30	12.00–32.00	65.65–72.00
	M—Subadult	15	50.6 (15.8)	52.0 [26.0, 80.0]			NA	NA	NA
	M—Adult	8	53.5 (13.1)	49.0 [40.0, 70.0]			NA	NA	NA
	Overall	429	47.11 (13.17)	46 [11, 110]			24–73.25	22–26	70.5–80
Hydroxybutyrate	F—Hatchling	17	0.91 (0.59)	0.67 [0.27, 2.30]	CBC	0.000–0.000	NA	NA	NA
mmol/L	F—Juvenile	56	0.66 (0.42)	0.57 [0.08, 1.95]			0.09–1.78	0.08–0.18	1.42–1.95
	F—Subadult	38	0.50 (0.37)	0.39 [0.10, 1.56]			0.08–1.62	0.06–0.12	1.20–2.12
	F—Adult	11	0.47 (0.37)	0.425 [0.07, 1.0]			NA	NA	NA
	M—Hatchling	71	1.10 (0.70)	0.92 [0.07, 2.76]			0.07–2.70	0.07–0.19	2.44–2.76
	M—Juvenile	82	0.83 (0.54)	0.74 [0.07, 2.79]			0.07–2.15	0.07–0.19	2.07–2.79
	M—Subadult	14	0.54 (0.46)	0.34 [0.07, 1.33]			NA	NA	NA
	M—Adult	8	0.34 (0.24)	0.275 [0.15, 0.75]			NA	NA	NA
	Overall	386	0.81 (0.56)	0.69 [0.07, 2.79]			0.08–2.26	0.07–0.15	2.10–2.45
A G ratio	F—Hatchling	22	0.3 (0.05)	0.28 [0.24, 0.41]	PE	0.001–0.000	0.23–0.45	0.22–0.24	0.37–0.58
	F—Juvenile	60	0.27 (0.03)	0.27 [0.21, 0.35]			0.21–0.35	0.21–0.22	0.33–0.35
	F—Subadult	39	0.24 (0.04)	0.23 [0.17, 0.31]			NA	NA	NA
	F—Adult	12	0.22 (0.04)	0.22 [0.16, 0.27]			NA	NA	NA
	M—Hatchling	74	0.3 (0.05)	0.29 [0.17, 0.43]			0.17–0.41	0.17–0.22	0.38–0.43
	M—Juvenile	86	0.27 (0.05)	0.27 [0.17, 0.37]			0.17–0.36	0.17–0.19	0.35–0.37
	M—Subadult	15	0.25 (0.05)	0.25 [0.19, 0.35]			NA	NA	NA
	M—Adult	8	0.25 (0.06)	0.24 [0.19, 0.34]			NA	NA	NA
	Overall	430	0.28 (0.05)	0.28c[0.16, 0.43]			0.18–0.38	0.17–0.19	0.37–0.40
Proportion of	F—Hatchling	22	0.23 (0.02)	0.22 [0.19, 0.29]	PE	0.001–0.000	0.19–0.31	0.18–0.19	0.27–0.37
albumin	F—Juvenile	60	0.21 (0.02)	0.21 [0.17, 0.26]			0.17–0.25	0.17–0.18	0.24–0.26
	F—Subadult	39	0.19 (0.03)	0.19 [0.14, 0.24]			0.14–0.24	0.13–0.15	0.23–0.25
	F—Adult	12	0.18 (0.02)	0.18 [0.14, 0.21]			NA	NA	NA
	M—Hatchling	74	0.23 (0.03)	0.22 [0.14, 0.29]			0.14–0.29	0.14–0.18	0.28–0.29
	M—Juvenile	86	0.21 (0.03)	0.21 [0.14, 0.27]			0.15–0.26	0.14–0.16	0.26–0.27
	M—Subadult	15	0.2 (0.03)	0.19 [0.16, 0.26]			NA	NA	NA
	M—Adult	8	0.2 (0.03)	0.19 [0.16, 0.25]			NA	NA	NA
	Overall	430	0.22 (0.03)	0.22 [0.14, 0.29]			0.15–0.28	0.14–0.16	0.27–0.28
Proportion of	F—Hatchling	22	0.01 (0.01)	0.01 [0.00, 0.03]	PE	0.000–0.000	0.00–0.04	0.00–0.00	0.03–0.05
Alpha-1 Globulins	F—Juvenile	60	0.01 (0.0)	0.01 [0.0, 0.04]			0.00–0.03	0.00–0.01	0.02–0.04
	F—Subadult	39	0.01 (0.0)	0.01 [0, 0.03]			NA	NA	NA
	F—Adult	12	0.0 (0.0)	0.0 [0.0, 0.01]			NA	NA	NA
	M—Hatchling	74	0.02 (0.01)	0.02 [0.0, 0.04]			0.00–0.04	0.00–0.01	0.03–0.04
	M—Juvenile	86	0.01 (0.0)	0.01 [0, 0.03]			0.00–0.03	0.00–0.00	0.03–0.03
	M—Subadult	15	0.01 (0.0)	0.01 [0.0, 0.02]			NA	NA	NA
	M—Adult	8	0.0 (0.0)	0.0 [0.0, 0.02]			NA	NA	NA
	Overall	430	0.01 (0.01)	0.01 [0.0, 0.05]			0.0–0.03	0.0–0.0	0.03–0.04
Alpha-1 Globulins	F—Hatchling	22	0.42 (0.43)	0.30 [0.01, 1.6]	PE	0.000–0.017	0.01–2.23	0.00–0.03	1.29–3.30
g/L	F—Juvenile	59	0.47 (0.38)	0.40 [0.0, 2.10]			0.01–1.70	0.00–0.05	1.10–2.10
	F—Subadult	39	0.40 (0.44)	0.20 [0, 1.80]			NA	NA	NA
	F—Adult	12	0.37 (0.20)	0.40 [0.09, 0.70]			NA	NA	NA
	M—Hatchling	74	0.75 (0.44)	0.70 [0.03, 1.80]			0.06–1.80	0.03–0.08	1.54–1.80
	M—Juvenile	86	0.57 (0.46)	0.50 [0, 2.0]			0.01–1.78	0.00–0.06	1.53–2.00
	M—Subadult	15	0.35 (0.25)	0.30 [0.04, 0.70]			NA	NA	NA
	M—Adult	8	0.39 (0.28)	0.35 [0.02, 0.90]			NA	NA	NA
	Overall	429	0.51 (0.41)	0.40 [0.0, 2.10]			0.01–1.63	0.01–0.02	1.50–1.80
Proportion of Gamma Globulins	F—Hatchling	22	0.0961 (0.0324)	0.085 [0.057, 0.162]	PE	0.000–0.000	0.05–0.20	0.04–0.06	0.14–0.26
	F—Juvenile	60	0.110 (0.0322)	0.104 [0.062, 0.226]			0.06–0.22	0.06–0.07	0.16–0.23
	F—Subadult	39	0.108 (0.0226)	0.106 [0.056, 0.153]			0.06–0.15	0.05–0.07	0.14–0.16
	F—Adult	12	0.128 (0.0275)	0.128 [0.092, 0.177]			NA	NA	NA
	M—Hatchling	74	0.0851 (0.0343)	0.079 [0.043, 0.18]			0.04–0.17	0.04–0.05	0.16–0.18
	M—Juvenile	86	0.100 (0.0290)	0.0965 [0.051, 0.186]			0.06–0.18	0.05–0.06	0.16–0.19
	M—Subadult	15	0.119 (0.0306)	0.114 [0.07, 0.169]			NA	NA	NA
	M—Adult	8	0.124 (0.0228)	0.124 [0.096, 0.168]			NA	NA	NA
	Overall	430	0.10 (0.03)	0.10 [0.04, 0.23]			0.05–0.17	0.05–0.05	0.16–0.18

Size class was determined by total length (hatchling ≤ 65 cm, juvenile 65 ≤ 150 cm, subadult 151 ≤ 225cm, and adult ≥ 225cm) as recommended by Mazzotti (17). Reference intervals were estimated based on non-parametric analysis except of those groups with insufficient samples (N < 40), from which we estimated reference intervals using robust Box–Cox transformed analysis. In the cases when we had an insufficient number of samples to obtain reference or confidence intervals (NA), we estimated overall (including all data) RIs. It is important to notice that these overall RIs are likely affected by size class and sex (see results) so it should be used with caution. Aberrant values were excluded from analysis as recommended by Friedrichs et al. (14) and are reported at the bottom of the table for reference. Abs, absolute count; SD, Standard Deviation; Cat, Category; CBC, complete blood count; CHEM, Chemistry; PE, Protein electrophoresis; M, male; F, female; SC, size class.

Aberrant values found in lymphocytes absolute counts females hatchling = 19.25 10^9^/L and subadult = 22.92 10^9^/L; AST females hatchling = 6.13 ukat/L, juvenile = 2.69 ukat/L, and subadults = 2.27 ukat/L; males hatchlings = 4.6 ukat/L and juveniles = 2.8 ukat/L; eosinophils absolute counts females juveniles = 5.77 10^9^/L, subadults = 9.59 10^9^/L, and adults = 10.43 10^9^/L, males juveniles = 6.11 and 7.03 10^9^/L, and overall (sex undetermined) = 9.59 and 10.43 10^9^/L; basophils absolute counts females subadults = 5.73 10^9^/L, males hatchlings = 2.91 10^9^/L, and overall = 5.73 10^9^/L; alpha-1 globulin juvenile females = 2.8 g/L; Calcium female subadult = 4.7 mmol/L, and overall (sex undetermined) = 4.7, 4.8, 6.32, and 7.42 mmol/L; total protein female juvenile = 560 g/L; hydroxybutyrate males hatchlings = 5.88 mmol/L.

The analyte influenced by sex (azurophils %) was biased toward males. Five out of the 12 analytes influenced by size class (WBC, CPK, uric acid, bile acid, and pr alpha-2 globulins) had higher values in hatchlings gradually decreasing toward adults, whereas potassium and beta globulins pr and abs had the inverse pattern (higher in adults gradually decreasing toward hatchlings; Table 3). The remaining four analytes showed higher values in hatchlings and adults and lower in juveniles and subadults. When pairwise, hatchling was the most dissimilar group compared with the other size classes finding very strong-to-moderate evidence of an effect on 23 (hatchling—subadult), 22 (hatchling—juvenile), and 20 (hatchling—adult) analytes (Supplementary Table 2). We did find strong-to-moderate evidence that TL can be used as a regressor in linear functions when modeling most of the analytes influenced by size class [heterophils % (*p*-value = 0.01), CPK (0.000), potassium (0.002), glucose (0.05), uric acid (0.000), pr alpha-2 globulin (0.000), beta globulins pr and abs (0.000 both), and bile acids (0.000)]. However, models only described up to 7 % of the variation of the data. Finally, we found little-to-no evidence that total length can be used as a regressor in a linear function in the case of WBC (0.15), monocytes % (0.93), and heterophil abs (0.57).

When modeling analytes influenced by sex and size class, we found strong-to-moderate evidence that sex rather than TL can be used as a regressor for PCV and that TL rather than sex can be used as a regressor for eosinophils %, calcium, sodium, and pr gamma globulins. We also found that males TL can be used as a regressor when modeling lymphocytes %, alpha-1 globulin pr and abs, and hydroxybutyrate and that females TL can be used as a regressor when modeling eosinophil abs, basophil abs, chloride, and total protein. Finally, we found strong evidence that both sex and TL can be used as a regressor when modeling lymphocyte abs, AST, A/G ratio, and pr albumin (Supplementary Table 3). R-squared adjusted values showed that models can explain 29 and 25 % of the variation of the data in the case of eosinophils % and abs, respectively, up to 23 % of the variation in the case of sodium and AST, 21 and 22 % in the case of pr albumin and A/G ratio, respectively, and up to 18 % in the case of calcium. The remaining models only could explain up to 11 % of the variation of the data found in those analytes.

Intraindividual variation showed that most animals recaptured had an increase in WBC, RBC, lymphocytes % and abs, eosinophils % and abs, basophils abs, CPK, total protein, alpha-1 globulin pr and abs, and beta globulin abs through time. In contrast, analytes such as PCV, heterophils %, monocytes % and abs, AST, calcium, potassium, sodium, chloride, glucose, triglycerides, A/G ratio, albumin pr and abs, alpha-2 globulin pr and abs, and bile acids showed a decreasing tendency in most individuals recaptured between 2015 and 2021. Finally, we found no changes through time in analytes such as azurophil % and abs in most of the recaptured animals.

## Discussion

This study represents the most extensive and thorough hematological and biochemical analysis performed on wild American crocodiles across its entire range, providing baseline information for the species for the first time. To date, no other hematological or plasma biochemical data have been documented for either wild or captive the American crocodile populations using current techniques, limiting spatial comparisons. Dessauer (5) reported the only plasma biochemical values for American crocodiles collected in the mid-twentieth century (6, 7, 8, 9; Supplementary Table 4). However, lack of specific information regarding the methods used for such estimations limits comparisons. Further research across Central and South America is warranted to clearly define analytes natural variation across different latitudes/landscapes, so more refined reference intervals can be provided for the species.

Mean values in American crocodiles for WBC, eosinophils % and abs, and monocytes % were higher than those reported in Orinoco crocodiles (11, 32), freshwater crocodiles (32), mugger crocodiles (33), Nile crocodiles (10), and Morelet's crocodiles (34) both captive and wild (Supplementary Table 4). More frequent pathogen exposure across environments and inter/intraspecific competition/aggression could be the main factors that explain higher leucocyte values reported for American crocodiles in South Florida, fairly relating to the values reported in wild heterospecific (Nile crocodiles). Lymphocyte was the most frequent leukocyte in American crocodiles relating to reported values in Nile crocodiles (10), but also differing from other crocodylians, like Orinoco crocodiles, with predominant heterophils leukograms. Uric acid and sodium values were higher on average compared with other crocodile species, which could be related to habitat variation across species and due to the fact American crocodiles' dwell in brackish/marine areas (greater exposure to dehydration), whereas Orinoco, freshwater, mugger, and Morelet's crocodiles inhabit freshwater areas. It also was found that chloride values were higher compared with heterospecific except for captive mugger crocodiles (33). The only other study conducted on a coastal crocodile (saltwater crocodile—*C. porosus*) also showed high maximum values of uric acid compared with freshwater crocodiles but was conducted on farmed animals (35). However, no data were collected regarding sodium or chloride values. Further studies are warranted to determine how seasonality, feeding frequencies, habitat type, environmental conditions, and quality and variety of diet interact with biochemistries or hematology in American crocodiles.

Analytes such as PCV, RBC, glucose, pr albumin, AST, calcium, potassium, and triglycerides fall within the range until now reported in other species from Crocodylidae. However, we were unable to compare values reported in our study for CPK, A/G ratio, pr albumin, alpha-1 globulin pr and abs, alpha-2 globulin pr and abs, beta globulins pr and abs, gamma globulins pr and abs, corticosterone, hydroxybutyrate, and bile acids due to the lack of information. When comparing these analytes with a well-studied Alligatoridae, the American alligator (*Alligator mississippiensis*) (36), we found much higher values for American crocodiles in CPK, A/G ratio, pr albumin, alpha-1 globulin abs, alpha-2 globulin pr and abs, beta globulin abs, corticosterone, and hydroxybutyrate (Supplementary Table 5) and only pr alpha-1 globulin and pr beta globulin were higher in American alligators.

It is known that the type of habitat a species is living in influences its physiology and can be reflected in the hematological and plasma biochemical values (37). Then, it is expected that a widespread species such as the American crocodile inhabiting a variety of habitats (from fresh to marine waters) across the Americas shows some level of heterogeneity blood parameters across its range. However, large variations in analyte values must be carefully analyzed because they can derive from natural constraints or anthropogenic impacts. This is the main reason why we did not estimate RIs for five out of the 40 analytes in which we found strong evidence that the area where animals were caught (highly human-influenced—TP vs. medium-to-low human-influenced BBE, ENP) impacted their performance, due the lack of certainty of the type of effects habitat could have.

Turkey point power plant has been considered an important area in South Florida for American crocodile population recovery due to the increasing number of individuals present in the area since the 1970s (22) caused by the unintentional construction of nesting habitat across berms and canals (38). This area utilizes over 250 km of canal to cool reactors providing habitat close to freshwater and other natural resources as well as nesting and nursery refuges. In 2013, studies conducted by the University of Florida (17) detected system-wide changes in environmental conditions (temperature and salinity) believed to be linked to localized drought and algal blooms. As a result, declines in crocodile abundance and body condition were reported up to 2016. Since then, management to control these two environmental variables oscillating under normal levels has been in place and both population parameters have been going upward (17). Blood parameters analyzed in the present study showed that most of the analytes assessed do not differ considerably between less impacted areas (ENP and BBE) and TP, which could be read as an indicative of the current system recovery. However, analytes related to stress such as corticosterone, gamma globulin abs, and alpha-2 globulin abs significantly differ among areas, which might also indicate some unidentified stressor could still be affecting TP populations. It is important to notice there are no studies assessing the stability of American crocodile corticosterone at 80° C for long periods of time, so we are assuming no effect on post processing (see methods). Further studies should address this issue to clarify whether this could be a potential confounding effect.

Analytes intraspecific variation are commonplace along vertebrates fluctuating across age and sex because of biological development (39). However, no previous evidence has been presented in the case of American crocodiles or any other crocodylian in the literature. Size was the variable that influenced the greatest number of analytes when evaluated as categorical (12 analytes) being hatchlings the most different group (Supplementary Table 2). However, this number drops to nine when evaluated as a continuous variable (total length) showing that the way categories are defined (grouped) influenced the result. A similar case happened when analyzing differences in means among categories across size class and sex (17 analytes affected by these two variables) and as continuous or absence or presence of categorical effect (dummy variables), getting a more detailed understanding of the effects in the latter. Variation in analytes such as eosinophils %, sodium, AST, pr albumin, and A/G ratio can be explained up to 29 % based on TL or a combination of TL and sex (female and/or male) proving useful for health assessments. However, other intrinsic (e.g., metabolism) or extrinsic (e.g., habitat) variables should be included in the model, so we can get a better understanding of the behavior of these blood parameters.

This positive/negative relationship between analytes and size was also observed when analyzed at the intraindividual level, because most of the analytes varied across time likely influenced by age/size, with most of them decreasing over time. This trend has also been reported in other species of crocodiles (11) as well as in other reptiles (39). However, it is not clear how and when these analytes shift and what variables influence them (i.e., physiological, environmental). Therefore, further studies focusing on intraindividual variation through time including biological (i.e., age) and environmental (i.e., salinity) factors are required to clearly understand intrinsic/allochthonous influences and their effect on reference intervals. Finally, even though we collected samples as standardized as possible focusing on causing minimum distress on crocodiles (see methods), it is important to highlight that some potential confounding effects could derive from animals' manipulation (capture/handling/releasing), potentially influencing some of the metrics assessed. For instance, taking samples immediately after capture could likely be correlated with initial epinephrine surges, influencing some of the metrics assessed (i.e., glucose, leukocyte %). However, as the method used in the present study for capture and handling American crocodiles is the most common/broadly used across its range, our metrics should relate with what could be reported elsewhere.

## Data availability statement

The original contributions presented in the study are included in the article/Supplementary material, further inquiries can be directed to the corresponding author/s.

## Ethics statement

The animal study was reviewed and approved by University of Florida Institutional Animal Care and Use Committee (IACUC) protocol # 202109072.

## Author contributions

All authors listed have made a substantial, direct, and intellectual contribution to the work and approved it for publication.

## Funding

Funding and support for this project has been provided to the authors by the National Park Service (P0272508), United States Army Corps of Engineers (P0229175), United States Geological Survey (P0195051), United States Fish and Wildlife Service (P0276515), Florida Power and Light Co. (P0262194), and the Florida Fish and Wildlife Conservation Commission (P0098980).

## Conflict of interest

Author LB was employed by United States Fish and Wildlife Service. The remaining authors declare that the research was conducted in the absence of any commercial or financial relationships that could be construed as a potential conflict of interest.

## Publisher's note

All claims expressed in this article are solely those of the authors and do not necessarily represent those of their affiliated organizations, or those of the publisher, the editors and the reviewers. Any product that may be evaluated in this article, or claim that may be made by its manufacturer, is not guaranteed or endorsed by the publisher.

## Author disclaimer

The findings and conclusions in this article are those of the author(s) and do not necessarily represent the views of the United States Fish and Wildlife Service. Any use of trade, firm, or product names is for descriptive purposes and does not imply endorsement by the United States Government.
