# Supplementary material for: Hematology and biochemistry reference intervals for American crocodiles (Crocodylus acutus) in South Florida
Source: Front Vet Sci. 2022 Nov 22;9:919488. doi: 10.3389/fvets.2022.919488 (PMC9722956; doi:10.3389/fvets.2022.919488)
Supplement: Supplementary file 1 [file Data_Sheet_1.docx]

**Supplementary Table 1.** Total number of analytes assessed (40) on American crocodiles (*Crocodylus acutus*) captured across South Florida, United States from 2015 to 2021. Notice sample sizes and statistic critical values for normality (Shapiro Wilk test) and homoscedasticity (Fligner-Killeen test), for the whole dataset (N), by size class and by Sex. Nh = hatchling (TL ≤ 65 cm) sample size, Nj = juvenile (65 – 150 cm) sample size, Ns = subadult (151 - 225cm) sample size, Na = adult (≥ 225cm) sample size, Nf = female sample size, Nm = male sample size, abs = absolute counts.

| **Analyte** | **Overall** | | **Grouped by size class** | | | | | **Grouped by sex** | | |
| --- | --- | --- | --- | --- | --- | --- | --- | --- | --- | --- |
|  | **N** | **Shapiro Wilk** | **Nh** | **Nj** | **Ns** | **Na** | **Fligner-killeen** | **Nf** | **Nm** | **Fligner-killeen** |
| White Blood Cell Count 10^9^/L | 413 | 0.00 | 178 | 160 | 54 | 21 | 0.96 | 134 | 174 | 0.20 |
| Red Blood Cell Count 10^9^/L | 381 | 0.00 | 169 | 140 | 52 | 20 | 0.50 | 127 | 152 | 0.04 |
| PCV | 407 | 0.00 | 179 | 154 | 54 | 20 | 0.40 | 130 | 169 | 0.83 |
| Heterophils % | 422 | 0.00 | 184 | 163 | 54 | 21 | 0.01 | 134 | 178 | 0.00 |
| Lymphocytes % | 422 | 0.00 | 184 | 163 | 54 | 21 | 0.39 | 134 | 178 | 0.06 |
| Azurophil % | 421 | 0.00 | 183 | 163 | 54 | 21 | 0.48 | 134 | 178 | 0.00 |
| Monocytes % | 376 | 0.00 | 157 | 149 | 51 | 19 | 0.09 | 120 | 171 | 0.65 |
| Eosinophils % | 421 | 0.00 | 183 | 163 | 54 | 21 | 0.00 | 134 | 178 | 0.00 |
| Basophils % | 421 | 0.00 | 183 | 163 | 54 | 21 | 0.00 | 134 | 178 | 0.00 |
| Heterophils abs 10^9^/L | 376 | 0.00 | 157 | 149 | 51 | 19 | 0.14 | 120 | 171 | 0.01 |
| Lymphocyte abs 10^9^/L | 376 | 0.00 | 1069 | 1274 | 354 | 84 | 0.21 | 875 | 1389 | 0.28 |
| Azurophil abs 10^9^/L | 376 | 0.00 | 157 | 149 | 51 | 19 | 0.80 | 120 | 171 | 0.07 |
| Monocyte abs 10^9^/L | 376 | 0.00 | 157 | 149 | 51 | 19 | 0.90 | 120 | 171 | 0.33 |
| Eosinophil abs 10^9^/L | 376 | 0.00 | 157 | 149 | 51 | 19 | 0.00 | 120 | 171 | 0.00 |
| Basophil abs 10^9^/L | 375 | 0.00 | 157 | 149 | 50 | 19 | 0.00 | 119 | 171 | 0.00 |
| AST ukat/L | 428 | 0.00 | 190 | 164 | 54 | 20 | 0.00 | 133 | 181 | 0.00 |
| CPK ukat/L | 427 | 0.00 | 189 | 164 | 54 | 20 | 0.00 | 133 | 180 | 0.00 |
| Calcium mmol/L | 428 | 0.00 | 190 | 164 | 54 | 20 | 0.00 | 133 | 181 | 0.23 |
| Potassium mmol/L | 436 | 0.00 | 193 | 167 | 55 | 21 | 0.00 | 136 | 184 | 0.00 |
| Sodium mmol/L | 436 | 0.00 | 193 | 166 | 55 | 21 | 0.01 | 136 | 183 | 0.01 |
| Chloride mmol/L | 435 | 0.03 | 193 | 167 | 55 | 21 | 0.29 | 136 | 184 | 0.73 |
| Glucose mmol/L | 436 | 0.00 | 193 | 167 | 55 | 21 | 0.13 | 136 | 184 | 0.62 |
| Phosphorus mg/dL | 428 | 0.00 | 190 | 164 | 54 | 20 | 0.01 | 133 | 181 | 0.42 |
| Uric acid mg/dL | 436 | 0.00 | 193 | 167 | 55 | 21 | 0.62 | 136 | 184 | 0.28 |
| Total protein g/L | 430 | 0.00 | 192 | 164 | 54 | 20 | 0.04 | 133 | 183 | 0.35 |
| Triglycerides mmol/L | 434 | 0.00 | 192 | 167 | 54 | 21 | 0.00 | 135 | 184 | 0.06 |
| A/G ratio | 430 | 0.03 | 192 | 164 | 54 | 20 | 0.10 | 133 | 183 | 0.02 |
| Proportion of albumin | 430 | 0.24 | 192 | 164 | 54 | 20 | 0.42 | 133 | 183 | 0.01 |
| Albumin g/L | 430 | 0.00 | 192 | 164 | 54 | 20 | 0.72 | 133 | 183 | 0.17 |
| Proportion of Alpha.1 Globulin | 430 | 0.00 | 192 | 164 | 54 | 20 | 0.00 | 133 | 183 | 0.23 |
| Alpha.1 Globulin g/L | 430 | 0.00 | 192 | 164 | 54 | 20 | 0.05 | 133 | 183 | 0.01 |
| Proportion of Alpha.2 Globulin | 430 | 0.00 | 192 | 164 | 54 | 20 | 0.87 | 133 | 183 | 0.86 |
| Alpha.2 Globulin g/L | 430 | 0.00 | 192 | 164 | 54 | 20 | 0.00 | 133 | 183 | 0.00 |
| Proportion of Beta Globulin | 430 | 0.00 | 192 | 164 | 54 | 20 | 0.31 | 133 | 183 | 0.48 |
| Beta Globulin g/L | 430 | 0.00 | 192 | 164 | 54 | 20 | 0.00 | 133 | 183 | 0.00 |
| Proportion of Gamma Globulin | 430 | 0.00 | 192 | 164 | 54 | 20 | 0.46 | 133 | 183 | 0.28 |
| Gamma Globulin g/L | 430 | 0.00 | 192 | 164 | 54 | 20 | 0.00 | 133 | 183 | 0.00 |
| Corticosterone nmol/L | 386 | 0.00 | 163 | 152 | 52 | 19 | 0.03 | 122 | 176 | 0.20 |
| Hydroxybutyrate mmol/L | 387 | 0.00 | 164 | 152 | 52 | 19 | 0.00 | 122 | 176 | 0.00 |
| Bile acid µmol/L | 424 | 0.00 | 188 | 164 | 53 | 19 | 0.00 | 132 | 180 | 0.00 |

**Supplementary Table 2.** American crocodile (*Crocodylus acutus*) analyte pairwise analysis (Dunn test) between size classes based on the blood samples collected in South Florida, United States from 2015 to 2021. Notice in bold all the pairwise comparisons with very strong-to moderate evidence of effect. A-H = Adult – Hatchling, A-J = Adult – Juvenile, H-J = Hatchling – Juvenile, A-S = Adult – Subadult, H-S = Hatchling – Subadult, and J-S = Juvenile - Subadult

| **Analyte** | $\boldsymbol{X}^{\boldsymbol{2}}$ | **A-H** | **A-J** | **H-J** | **A-S** | **H-S** | **J-S** |
| --- | --- | --- | --- | --- | --- | --- | --- |
| White Blood Cell Count 10^9^/L | 22.71 | 1.00 | **0.02** | **0.00** | **0.18** | **0.13** | 0.92 |
| Red Blood Cell Count 10^9^/L | 6.06 | **0.24** | 0.54 | 1.00 | 1.00 | **0.14** | 0.54 |
| PCV | 30.65 | **0.08** | 1.00 | **0.00** | 1.00 | **0.00** | **0.47** |
| Heterophils % | 36.30 | 1.00 | **0.09** | **0.00** | **0.23** | **0.00** | 1.00 |
| Lymphocytes % | 22.10 | **0.00** | **0.00** | 0.98 | **0.15** | **0.08** | **0.01** |
| Azurophils % | 2.87 | 1.00 | 1.00 | **0.48** | 1.00 | 0.63 | 1.00 |
| Monocytes % | 9.74 | **0.31** | **0.03** | **0.12** | **0.06** | **0.48** | 1.00 |
| Eosinophils % | 125.70 | **0.00** | **0.08** | **0.00** | 1.00 | **0.00** | **0.00** |
| Heterophil abs 10^9^/L | 10.28 | 1.00 | 1.00 | **0.01** | 1.00 | **0.20** | 1.00 |
| Lymphocyte abs 10^9^/L | 34.76 | **0.01** | **0.00** | **0.00** | **0.04** | 1.00 | **0.01** |
| Azurophil abs 10^9^/L | 1.22 | 1.00 | 1.00 | 1.00 | 1.00 | 1.00 | 1.00 |
| Monocyte abs 10^9^/L | 2.79 | 0.71 | **0.40** | 1.00 | **0.40** | 1.00 | 1.00 |
| Eosinophil abs 10^9^/L | 121.38 | **0.00** | **0.20** | **0.00** | 1.00 | **0.00** | **0.00** |
| Basophil abs 10^9^/L | 22.59 | 0.63 | 1.00 | **0.00** | 0.54 | **0.00** | **0.38** |
| AST ukat/L | 155.97 | **0.00** | 0.55 | **0.00** | 1.00 | **0.00** | **0.27** |
| CPK ukat/L | 60.09 | **0.00** | **0.05** | **0.00** | 1.00 | **0.00** | **0.00** |
| Calcium mmol/L | 52.86 | **0.00** | **0.00** | **0.00** | **0.09** | **0.00** | **0.02** |
| Potassium mmol/L | 9.40 | **0.18** | **0.06** | 0.96 | 1.00 | **0.27** | **0.06** |
| Sodium mmol/L | 80.06 | **0.00** | **0.00** | **0.00** | 0.93 | **0.00** | **0.00** |
| Chloride mmol/L | 19.22 | **0.03** | **0.08** | 1.00 | 1.00 | **0.00** | **0.00** |
| Glucose mmol/L | 25.71 | 1.00 | **0.09** | **0.00** | **0.39** | **0.03** | 1.00 |
| Uric acid mg/dL | 20.34 | **0.01** | **0.12** | **0.06** | 1.00 | **0.00** | **0.16** |
| Total protein g/L | 49.04 | **0.00** | **0.07** | **0.00** | 0.93 | **0.00** | **0.27** |
| Triglycerides mmol/L | 2.60 | 0.58 | **0.48** | 1.00 | **0.34** | 1.00 | 1.00 |
| A/G ratio | 90.27 | **0.00** | **0.00** | **0.00** | 1.00 | **0.00** | **0.00** |
| Proportion of albumin | 87.60 | **0.00** | **0.00** | **0.00** | 1.00 | **0.00** | **0.00** |
| Albumin g/L | 4.05 | **0.26** | 0.82 | 0.53 | 0.82 | 1.00 | 1.00 |
| Proportion of Alpha.1 Globulin | 37.71 | **0.00** | **0.01** | **0.00** | **0.26** | **0.00** | **0.30** |
| Alpha.1 Globulin g/L | 10.24 | **0.50** | 0.98 | 1.00 | 1.00 | **0.01** | **0.05** |
| Proportion of Alpha.2 Globulin | 15.49 | **0.03** | **0.41** | **0.05** | 1.00 | **0.01** | **0.43** |
| Proportion of Beta Globulin | 45.60 | **0.00** | **0.35** | **0.00** | 1.00 | **0.00** | **0.00** |
| Beta Globulin g/L | 25.62 | **0.00** | **0.05** | **0.00** | **0.09** | **0.04** | 1.00 |
| Proportion of Gamma Globulin | 55.54 | **0.00** | **0.01** | **0.00** | **0.29** | **0.00** | **0.17** |
| Hydroxybutyrate mmol/L | 44.29 | **0.00** | **0.01** | **0.01** | 1.00 | **0.00** | **0.00** |
| Bile acid µmol/L | 61.23 | **0.00** | 0.89 | **0.00** | 1.00 | **0.00** | **0.04** |

**Supplementary Table 3.** American crocodile (*Crocodylus acutus*) multiple linear regression critical values obtained when modeling analytes affected by both sex and size class. Notice that even though we found strong evidence based on Kruskall-Wallis test (table 3) that both variables affected all the analytes described below, when modeling using linear regression based on continuous (total length) and dummy variables (Female/Male; see results) the effect of these variables differ across analytes. Pr = Proportion of, abs = Absolute counts.

| **Analytes** | **Female** | **Male** | **Total length** |
| --- | --- | --- | --- |
| PCV | **0.000** | **0.000** | 0.10 |
| Eosinophils % | 0.07 | 0.62 | **0.000** |
| Calcium | 0.22 | 0.68 | **0.000** |
| Sodium | 0.41 | 0.11 | **0.000** |
| pr gamma globulins | 0.42 | 0.65 | **0.000** |
| Lymphocytes % | 0.24 | **0.000** | **0.000** |
| pr alpha 1 globulin | 0.55 | **0.016** | **0.000** |
| Alpha 1 globulin abs | 0.06 | **0.000** | **0.004** |
| Hydroxybutyrate | 0.86 | **0.030** | **0.000** |
| Eosinophil abs | **0.01** | 0.44 | **0.000** |
| Basophil abs | **0.01** | 0.15 | **0.020** |
| Chloride | **0.014** | 0.17 | **0.040** |
| Total protein | **0.019** | 0.33 | **0.028** |
| Lymphocyte abs | **0.001** | **0.000** | **0.026** |
| AST | **0.000** | **0.000** | **0.000** |
| A/G ratio | **0.01** | **0.04** | **0.000** |
| pr albumin | **0.009** | **0.02** | **0.000** |

**Supplementary Table 4.** Hematological and biochemical general statistics (mean, SD = standard deviation, SE = standard error, median, Min = minimum, Max = maximum) for American crocodiles (*Crocodylus acutus*) and heterospecific (Orinoco crocodiles – *C. intermedius*, freshwater crocodiles - *C. johnstoni*, saltwater crocodiles - *C. porosus*, mugger crocodiles - *C. palustris*, Nile crocodiles – *C. niloticus*, and morelet’s crocodiles – *C. moreletti*) reported in this study and in literature. Units are presented consistently as reported in the original literature, so values are not present under the international standard. In the case of the five analytes in which we found strong evidence of an area effect (marked with an *), we present general statistics of those collected on low-medium human impacted areas (Everglades National Park, Florida, USA and Biscayne Bay Estuary, Florida, USA).

| **Analyte** | **Our Study** | | ***C. acutus* (a)** | | ***C. intermedius* captive, n= 326) (b)** | | ***C. intermedius* (Juv) Wild (n= 80) (c)** | | ***C. johnstoni* Wild (n= 39) (d)** | | ***C. porosus* Captive (n= 39) (e)** | | ***C. palustris* Juv. Captivity (n= 24) (f)** | |
| --- | --- | --- | --- | --- | --- | --- | --- | --- | --- | --- | --- | --- | --- | --- |
|  | **Mean (SD)** | **Median [Min-Max]** | **Mean** | **Min-Max** | **Mean ± SD** | **Min-Max** | **Mean ± SD** | **Min-Max** | **Mean ± SE** | **Min-Max** | **Mean** | **Min-Max** | **Mean ± SD** | **Min-Max** |
| PCV (%) | 19 (5.0) | 19 [5, 38] | - | - | 26.3 ± 3.7 | 17 - 36 | 24.7 ± 2.5 | - | 23.1 ± 3.4 | 18.0 - 32.0 | - | 17 - 41 | 24.9 ± 5.38 | 16 - 38 |
| RBC (10^6^/μl) | 0.76 (0.30) | 0.75 [0.10, 1.87] | - | - | 1.1 ± 0.4 | 0.47 - 2.28 | - | - | - | - | - | 0.6 - 1.3 | 0.69 ± 0.06 | 0.58 - 0.81 |
| WBC (10^3^/μl) | 12.6 (4.72) | 12.0 [2.00, 33.7] | - | - | 6.8 ± 2.5 | 2.2 - 15.1 | 6.60 ± 1.0 | - | 8.0 ± 3.9 | 2.2 - 17.5 | - | 6.4 - 25.7 | - | - |
| Heterophils (%) | 24.0 (19.4) | 18.0 [1.00, 88.0] | - | - | 60.8 ± 12.1 | 20 - 83 | 55.8 ± 8.8 | - | - | - | - | - | - | - |
| Heterophils (10^3^/μl) | 2.63 (2.66) | 1.86 [0, 20.6] | - | - | 4.10 ± 1.94 | 0.3 - 9.5 | - | - | 1.3 ± 1.3 | 0.1 - 6.8 | - | 0.8 - 7.4 | 5.60 ± 1.58 | 3.12 - 9.55 |
| Lymphocytes (%) | 58.7 (19.4) | 62.0 [6.0, 96.0] | - | - | 27.2 ± 10.5 | Jun-65 | 31.8 ± 10.2 | - | - | - | - | - | - | - |
| Lymphocytes (10^3^/μl) | 7.4 (3.81) | 7.02 [0, 22.9] | - | - | 1.72 ± 0.74 | 0.3 - 3.6 | - | - | 5.1 ± 2.5 | 1.4 - 9.8 | - | 4.5 - 21.6 | 2.48 ± 1.02 | 1.16 - 4.93 |
| Eosinophils (%) | 8.54 (8.99) | 6.00 [0, 64.0] | - | - | 3.6 ± 3.3 | 0 - 16 | 8.3 ± 5.6 | - | - | - | - | - | - | - |
| Eosinophils (10^3^/μl) | 1.11 (1.32) | 0.680 [0, 10.4] | - | - | 0.18 ± 0.15 | 0 - 0.62 | - | - | 0.7 ± 0.5 | 0.0 - 1.6 | - | 0.0 - 0.7 | 0.53 ± 0.27 | 0.0 - 1.29 |
| Monocytes (%) | 3.57 (3.81) | 2.00 [0, 23.0] | - | - | 1.7 ± 3.4 | 0 - 20 | 1.6 ± 1.9 | - | - | - | - | - | - | - |
| Monocytes (10^3^/μl) | 0.436 (0.544) | 0.280 [0, 5.47] | - | - | 0.05 ± 0.085 | 0 - 0.34 | - | - | 1.0 ± 0.8 | 0.0 - 2.7 | - | 0.0 - 1.2 | 0.09 ± 0.09 | 0 - 0.32 |
| Basophils (%)* | 5.04 (4.45) | 4.00 [0, 21.0] | - | - | 6.6 ± 4.2 | 0 - 20 | 3.0 ± 2.1 | - | - | - | - | - | - | - |
| Basophils (10^3^/μl) | 0.654 (0.662) | 0.490 [0, 5.73] | - | - | 0.40 ± 0.24 | 0 - 1.09 | - | - | 0.0 ± 0.1 | 0.0 - 0.6 | - | 0.0 - 0.7 | 0.01 ± 0.03 | 0 - 0.10 |
| Azurophils (%) | 0.582 (1.94) | 0 [0, 18.0] | - | - | 0.0 ± 0.2 | 0 - 3 | - | - | - | - | - | - | - | - |
| Azurophils (10^3^/μl) | 0.0182 (0.137) | 0 [0, 2.04] | - | - | 0.001 ± 0.001 | 0 - 0.28 | - | - | - | - | - | - | - | - |
| UA (mg/dl) | 7.51 (4.39) | 6.50 [0.500, 36.5] | - | - | 4.4 ± 1.7 | 1.8 - 7.9 | - | - | 1.1 ± 0.42 | 0.4 - 1.89 | - | 2.8 - 16.6 | 3.86 ± 1.3 | 2.4 - 7.0 |
| Glucose (mg/dl) | 69.4 (17.9) | 68.0 [28.0, 125] | 101 | - | 128.1 ± 23.5 | 92 - 186 | - | - | 64.8 ± 18 | 36.0 - 111 | - | 81.0 - 217 | 64.88 ± 13.95 | 48 - 97 |
| ALB (g/dl) | 1.04 (0.595) | 1.00 [0.180, 11.8] | - | - | 2.0 ± 0.3 | 1.4 - 3 | - | - | 0.77 ± 0.3 | 0.0 - 1.5 | - | 1.4 - 2.3 | 1.14 ± 0.12 | 1.0 - 1.4 |
| AST (U/l) | 66.7 (43.7) | 55.0 [12.0, 367] | - | - | 103.3 ± 64.6 | 25 - 260 | - | - | 36.5 ± 12.8 | 19.0 - 74.0 | - | 23 - 157 | 52.13 ± 8.88 | 36 - 70 |
| CPK U/L | 5390 (6920) | 1940 [45.0, 54900] | - | - | - | - | - | - | - | - | - |  | - | - |
| Calcium (mg/dL) | 12.2 (1.69) | 12.1 [8.50, 29.7] | 3.4 | - | - | - | - | - | 2.3 ± 0.5 | 1.0–2.9 | - | 2.41 - 3.45 | 12.72 ± 0.51 | 11.9 - 13.5 |
| Potassium (mmol/L) | 4.93 (0.997) | 4.80 [2.80, 8.80] | 7.9 | - | - | - | - | - | 3.7 ± 0.8 | 2.3–5.2 | - | 3.8 - 7.2 | 8.0 ± 0.4 | 7.3 - 8.9 |
| Sodium (mmol/L) | 150 (9.99) | 148 [130, 184] | 149 | - | - | - | - | - | 138.5 ± 9.7 | 111–154 | - | 143 - 161 | 143.17 ± 6.15 | 133 - 166 |
| Chloride (mmol/L) | 120 (9.01) | 120 [91.0, 151] | 117 | - | - | - | - | - | - | - | - | 88 - 127 | 119.71 ± 9.69 | 98 - 139 |
| Phosphorus (mg/dL)* | 5.72 (1.53) | 5.60 [1.60, 12.3] | - | - | - | - | - | - | 1.2 ± 0.4 | 0.5–1.9 | - | 1.2 - 2.9 | 5.06 ± 1.12 | 4.2 - 9.6 |
| Total Proteins (g/dL) | 4.83 (2.80) | 4.60 [1.10, 56.0] | - | - | - | - | - | - | 39.6 ± 14.9 | 15.0–70.0 | - | 41 - 70 | 3.12 ± 0.23 | 2.0 - 4.1 |
| Triglycerides (mg/dL) | 110 (150) | 58.5 [5.00, 1420] | - | - | - | - | - | - | - | - | - | 0.1 - 8.8 | 390.42 ± 326.63 | (36 - 1,331) |
| A/G ratio | 0.281 (0.0510) | 0.280 [0.160, 0.430] | - | - | - | - | - | - | - | - | - |  | - | - |
| Albumin (%) | 21.6 (3.09) | 21.6 [13.7, 29.3] | - | - | - | - | - | - | - | - | - |  | - | - |
| Alpha 1 Globulins (%) | 1.45 (0.857) | 1.40 [0, 5.40] | - | - | - | - | - | - | - | - | - |  | - | - |
| Alpha 1 Globulins (g/dL) | 0.0658 (0.0400) | 0.0600 [0, 0.280] | - | - | - | - | - | - | - | - | - |  | - | - |
| Alpha 2 Globulins (%) | 24.5 (3.70) | 24.2 [13.8, 41.1] | - | - | - | - | - | - | - | - | - |  | - | - |
| Alpha 2 Globulins (g/dL)* | 1.19 (0.659) | 1.13 [0.240, 12.2] | - | - | - | - | - | - | - | - | - |  | - | - |
| Beta Globulins (%) | 42.3 (4.58) | 42.3 [25.1, 63.1] | - | - | - | - | - | - | - | - | - |  | - | - |
| Beta Globulins (g/dL) | 2.05 (1.37) | 1.96 [0.450, 27.4] | - | - | - | - | - | - | - | - | - |  | - | - |
| Gamma Globulins (%) | 9.92 (3.24) | 9.60 [3.70, 22.6] | - | - | - | - | - | - | - | - | - |  | - | - |
| Gamma Globulins (g/dL)* | 0.477 (0.282) | 0.431 [0.0900, 4.31] | - | - | - | - | - | - | - | - | - |  | - | - |
| Corticosterone (ng/mL)* | 15.3 (15.5) | 9.63 [0.300, 98.6] | - | - | - | - | - | - | - | - | - |  | - | - |
| Hydroxybutyrate (mmol/L) | 0.824 (0.616) | 0.690 [0.0700, 5.88] | - | - | - | - | - | - | - | - | - |  | - | - |
| Bile acid | 11.5 (11.6) | 8.30 [0, 102] | - | - | - | - | - | - | - | - | - |  |  |  |

(a) *Crocodylus acutus* (Blood Chemistry of Reptiles Dessauer, W. R. 1970. Pp. 1–72.)

(b) Captive *Crocodylus intermedius* (Barajas-Valero et al. 2021)

(c) Wild juvenile *Crocodylus intermedius*, (Manzanilla et al. 2011, 360–356)

(d) Wild *Crocodylus johnstoni* (Scheelings et al. 2016, 959–961)

(e) Captive *Crocodylus porosus* (Millan et al. 1997, 814–817)

(f) Captive *Crocodylus palustris* (Stacy and Whitaker 2000, 339–347)

(g) Wild and captive *Crocodylus niloticus* (Lovely et al. 2007, 137–144)

(h) Wild and captive *Crocodylus moreletti* (Padilla et al. 2011, 511–522)

**Continue Supplementary Table 4.** Hematological and biochemical general statistics (mean, SD = standard deviation, SE = standard error, median, Min = minimum, Max = maximum) for American crocodiles (*Crocodylus acutus*) and heterospecific (Orinoco crocodiles – *C. intermedius*, freshwater crocodiles - *C. johnstoni*, saltwater crocodiles - *C. porosus*, mugger crocodiles - *C. palustris*, Nile crocodiles – *C. niloticus*, and morelet’s crocodiles – *C. moreletti*) reported in this study and in literature. Units are presented consistently as reported in the original literature, so values are not present under the international standard. In the case of the five analytes in which we found strong evidence of an area effect (marked with an *), we present general statistics of those collected on low-medium human impacted areas (Everglades National Park and Biscayne Bay Estuary).

| **Analyte** | **Our Study** | | ***C. palustris* sub adult Captivity (n= 16) (f)** | | ***C. palustris* adult Captivity (n= 14) (f)** | | ***C. niloticus* Wild (n= 38) (g)** | | ***C. niloticus* Captive (n= 44) (g)** | | ***C. moreletti* Captive (n= 47) (h)** | | ***C. moreletti* Wild (n= 45) (h)** | |
| --- | --- | --- | --- | --- | --- | --- | --- | --- | --- | --- | --- | --- | --- | --- |
|  | **Mean (SD)** | **Median [Min-Max]** | **Mean ± SD** | **Min-Max** | **Mean ± SD** | **Min-Max** | **Mean** | **Min-Max** | **Mean** | **Min-Max** | **Mean** | **Min-Max** | **Mean** | **Min-Max** |
| PCV (%) | 19 (5.0) | 19 [5, 38] | 22.45 ± 4.08 | 17 - 29 | 25.54 ± 2.54 | 19 - 30 | 17.9 | 14 - 22 | 27.2 | 24 - 31 | 24.5 | 23.2 - 25.7 | 24.6 | 22.7 - 26.4 |
| RBC (10^6^/μl) | 0.76 (0.30) | 0.75 [0.10, 1.87] | 0.71 ± 0.14 | 0.48 - 0.95 | 0.80 ± 0.12 | (0.64 - 0.99) | 0.59 | 0.35 - 1 | 0.92 | 0.6 - 1.31 | 1.1 | 1.02 - 1.17 | 1.04 | 0.92 - 1.16 |
| WBC (10^3^/μl) | 12.6 (4.72) | 12.0 [2.00, 33.7] | - | - | - | - | 11.28 | 3.75 - 26.22 | 6.4 | 4.0 - 11.5 | 8.3 | 8.0 - 8.5 | 9.9 | 9.4 - 10.4 |
| Heterophils (%) | 24.0 (19.4) | 18.0 [1.00, 88.0] | - | - | - | - | 20.5 | 4 - 39 | 13.4 | 6 -. 20 | - | - | - | - |
| Heterophils (10^3^/μl) | 2.63 (2.66) | 1.86 [0, 20.6] | 5.15 ± 1.71 | 3.33 - 9.72 | 4.45 ± 1.24 | 2.24 - 6.70 | 2.09 | 0.45 - 3.66 | - | - | 2.27 | 2.21 - 2.42 | 2.96 | 2.68 - 3.24 |
| Lymphocytes (%) | 58.7 (19.4) | 62.0 [6.0, 96.0] | - | - | - | - | 62 | 44 - 85 | 82.2 | 73 - 95 | - | - | - | - |
| Lymphocytes (10^3^/μl) | 7.4 (3.81) | 7.02 [0, 22.9] | 3.01 ± 1.11 | 1.2 - 4.93 | 1.83 ± 0.64 | 0.79 - 3.10 | 7.2 | 1.65 - 17.83 | - | - | 3.93 | 3.73 - 4.14 | 4.33 | 4.06 - 4.59 |
| Eosinophils (%) | 8.54 (8.99) | 6.00 [0, 64.0] | - | - | - | - | 4.9 | 0 - 17 | 4.4 | 2 -. 8 | - | - | - | - |
| Eosinophils (10^3^/μl) | 1.11 (1.32) | 0.680 [0, 10.4] | 0.33 ± 0.17 | 0.15 - 0.6 | 0.56 ± 0.24 | 0.14 - 1.02 | 0.53 | 0 - 2.14 | - | - | 0.26 | 0.21 - 0.32 | 0.26 | 0.21 - 0.33 |
| Monocytes (%) | 3.57 (3.81) | 2.00 [0, 23.0] | - | - | - | - | 0.9 | 0 - 10 | 2.5 | 1 -. 7 | - | - | - | - |
| Monocytes (10^3^/μl) | 0.436 (0.544) | 0.280 [0, 5.47] | 0.09 ± 0.08 | 0 - 0.26 | 0.09 ± 0.08 | 0.12 - 0.11 | 0.09 | 0 - 0.79 | - | - | 0.08 | 0.05 - 0.11 | 0.28 | 0.22 - 0.33 |
| Basophils (%)* | 5.04 (4.45) | 4.00 [0, 21.0] | - | - | - | - | 5.9 | 0 - 16 | - | - | - | - | - | - |
| Basophils (10^3^/μl) | 19 (5.0) | 19 [5, 38] | 0.03 ± 0.07 | 0 - 0.17 | 0.0 ± 0.0 | 0 | 0.69 | 0 - 2.9 | - | - | 1.5 | 1.44 - 1.72 | 1.8 | 1.62 - 1.92 |
| Azurophils (%) | 0.582 (1.94) | 0 [0, 18.0] | - | - | - | - | 5.1 | 0 - 21 | - | - | - | - | - | - |
| Azurophils (10^3^/μl) | 0.0182 (0.137) | 0 [0, 2.04] | - | - | - | - | 0.6 | 0 - 3.93 | - | - | 0.15 | 0.11 - 0.18 | 0.26 | 0.19 - 0.33 |
| UA (mg/dl) | 7.51 (4.39) | 6.50 [0.500, 36.5] | 3.86 ± 1.3 | 2.4 - 7.0 | 4.41 ± 1.38 | 2.2 - 7.7 | 2.16 | 0.67 - 5.04 | 4.03 | - | 1.95 | 1.51 - 2.33 | 4.48 | 3.13 - 5.84 |
| Glucose (mg/dl) | 69.4 (17.9) | 68.0 [28.0, 125] | 58.3 ± 7.23 | 50 - 74 | 76.79 ± 17.22 | 55 - 110 | 68.4 | 32.4 - 86.4 | 82.3 | - | 64.3 | 58.9 - 69.7 | 77.7 | 67.4 - 88.1 |
| ALB (g/dl) | 1.04 (0.595) | 1.00 [0.180, 11.8] | 1.11 ± 0.14 | 0.9 - 1.3 | 1.18 ± 0.11 | 1.0 - 1.4 | 1.4 | 1.1 - 1.9 | 1.9 | - | - | - | - | - |
| AST (U/l) | 66.7 (43.7) | 55.0 [12.0, 367] | 50.94 ± 9.12 | 30 - 65 | 41 ± 11.72 | 23 - 55 | 66.5 | 14 - 211 | 16.6 | - | - | - | - | - |
| CPK U/L | 5390 (6920) | 1940 [45.0, 54900] |  |  |  |  |  |  | - | - | - | - | - | - |
| Calcium (mg/dL) | 12.2 (1.69) | 12.1 [8.50, 29.7] | 12.76 ± 0.49 | (11.8 - 13.6) | 13.05 ± 0.31 | 12.8 - 14.0 | 49.14 |  | - | - | - | - | - | - |
| Potassium (mmol/L) | 4.93 (0.997) | 4.80 [2.80, 8.80] | 7.12 ± 0.69 | (5.9 - 8.2) | 7.79 ± 0.53 | 6.9 - 9.1 | 4.88 | 2.34 - 3.15 | - | - | - | - | - | - |
| Sodium (mmol/L) | 150 (9.99) | 148 [130, 184] | 144.44 ± 8.37 | (128 - 163) | 141.07 ± 6.86 | 133 - 161 | 147.9 | 122 - 164 | - | - | - | - | - | - |
| Chloride (mmol/L) | 120 (9.01) | 120 [91.0, 151] | 118.44 ± 2.42 | (113 - 122) | 188.86 ± 1.61 | 113 - 122 | 120.3 | 97 - 135 | - | - | - | - | - | - |
| Phosphorus (mg/dL)* | 5.72 (1.53) | 5.60 [1.60, 12.3] | 4.98 ± 0.54 | (4.5 - 6.9) | 4.99 ± 0.81 | 4.1 - 6.8 |  |  | - | - | - | - | - | - |
| Total Proteins (g/dL) | 4.83 (2.80) | 4.60 [1.10, 56.0] | 3.07 ± 0.22 | (2.7 - 3.5) | 3.19 ± 0.26 | 2.9 - 3.9 |  |  | - | - | - | - | - | - |
| Triglycerides (mg/dL) | 110 (150) | 58.5 [5.00, 1420] | 32.56 ± 7.19 | (21 - 46) | 49.5 ± 19.61 | 20 - 81 |  |  | - | - | - | - | - | - |
| A/G ratio | 0.281 (0.0510) | 0.280 [0.160, 0.430] |  |  |  |  |  |  | - | - | - | - | - | - |
| Albumin (%) | 21.6 (3.09) | 21.6 [13.7, 29.3] |  |  |  |  |  |  | - | - | - | - | - | - |
| Alpha 1 Globulins (%) | 1.45 (0.857) | 1.40 [0, 5.40] | - | - |  |  |  |  | - | - | - | - | - | - |
| Alpha 1 Globulins (g/dL) | 0.0658 (0.0400) | 0.0600 [0, 0.280] | - | - |  |  |  |  | - | - | - | - | - | - |
| Alpha 2 Globulins (%) | 24.5 (3.70) | 24.2 [13.8, 41.1] | - | - |  |  |  |  | - | - | - | - | - | - |
| Alpha 2 Globulins (g/dL)* | 1.19 (0.659) | 1.13 [0.240, 12.2] | - | - |  |  |  |  | - | - | - | - | - | - |
| Beta Globulins (%) | 42.3 (4.58) | 42.3 [25.1, 63.1] | - | - |  |  |  |  | - | - | - | - | - | - |
| Beta Globulins (g/dL) | 2.05 (1.37) | 1.96 [0.450, 27.4] | - | - |  |  |  |  | - | - | - | - | - | - |
| Gamma Globulins (%) | 9.92 (3.24) | 9.60 [3.70, 22.6] | - | - |  |  |  |  | - | - | - | - | - | - |
| Gamma Globulins (g/dL)* | 0.477 (0.282) | 0.431 [0.0900, 4.31] | - | - |  |  |  |  | - | - | - | - | - | - |
| Corticosterone (ng/mL)* | 15.3 (15.5) | 9.63 [0.300, 98.6] | - | - |  |  |  |  | - | - | - | - | - | - |
| Hydroxybutyrate (mmol/L) | 0.824 (0.616) | 0.690 [0.0700, 5.88] | - | - |  |  |  |  | - | - | - | - | - | - |
| Bile acid | 11.5 (11.6) | 8.30 [0, 102] |  |  |  |  |  |  | - | - | - | - | - | - |

**Supplementary Table 5.** Hematological and biochemical general statistics for American crocodiles (*Crocodylus acutus*) and American alligators (*Alligator mississppiensis*) reported by Brandt (38). Units are presented consistently as reported in the original literature, so values are not present under the international standard. SD = standard deviation, Min = minimum, Max = maximum.

| **Analyte** | **Our Study** | | ***Alligator mississippiensis* wild (n= 120)** | |
| --- | --- | --- | --- | --- |
|  | **Mean (SD)** | **Median [Min-Max]** | **Mean** | **Reference interval** |
| PCV (%) | 19 (5.0) | 19 [5, 38] | 23.81 | 12.44 – 35.21 |
| RBC (10^6^/μl) | 0.76 (0.30) | 0.75 [0.10, 1.87] | 0.81 | 0.37 – 1.71 |
| WBC (10^3^/μl) | 12.6 (4.72) | 12.0 [2.00, 33.7] | 13.21 | 5.25 – 32.42 |
| Heterophils (%) | 24.0 (19.4) | 18.0 [1.00, 88.0] | 22.36 | 9.72 – 55.77 |
| Heterophils (10^3^/μl) | 2.63 (2.66) | 1.86 [0, 20.6] | 2.75 | 0.76 – 8.33 |
| Lymphocytes (%) | 58.7 (19.4) | 62.0 [6.0, 96.0] | 35.88 | 4.70 – 67.67 |
| Lymphocytes (10^3^/μl) | 7.4 (3.81) | 7.02 [0, 22.9] | 4.21 | 0.41 – 13.50 |
| Eosinophils (%) | 8.54 (8.99) | 6.00 [0, 64.0] | 2.67 | 0.0072 – 13.86 |
| Eosinophils (10^3^/μl) | 1.11 (1.32) | 0.680 [0, 10.4] | 0.33 | 0.00 – 2.21 |
| Monocytes (%) | 3.57 (3.81) | 2.00 [0, 23.0] | 24.25 | 4.32 – 44.57 |
| Monocytes (10^3^/μl) | 0.436 (0.544) | 0.280 [0, 5.47] | 3.1 | 0.43 – 8.16 |
| Basophils (%) | 5.04 (4.45) | 4.00 [0, 21.0] | 7.98 | 1.62 – 18.80 |
| Basophils (10^3^/μl) | 0.64 (0.61) | 0.49 [0, 2.91] | 0.97 | 0.12 – 2.62 |
| Azurophils (%) | 0.582 (1.94) | 0 [0, 18.0] |  |  |
| Azurophils (10^3^/μl) | 0.01 (0.05) | 0 [0, 0.47] |  |  |
| Uric Acid (mg/dl) | 7.51 (4.39) | 6.50 [0.50, 36.5] | 1.23 | 0.61 – 2.37 |
| Glucose (mg/dl) | 69.4 (17.9) | 68.0 [28.0, 125] | 72.25 | 41.30 – 124.69 |
| ALB (g/dl) | 1.04 (0.595) | 1.00 [0.180, 11.8] | 0.71 | 0.44 – 1.10 |
| AST (U/l) | 66.7 (43.7) | 55.0 [12.0, 367] | 201.65 | 106.52 – 292.57 |
| CPK U/L | 5390 (6920) | 1940 [45.0, 54900] | 1316.64 | 286.23 – 5230.34 |
| Calcium (mg/dL) | 12.2 (1.69) | 12.1 [8.50, 29.7] | 11.61 | 9.49 – 13.67 |
| Potassium (mmol/L) | 4.93 (0.997) | 4.80 [2.80, 8.80] | 4.93 | 3.14 – 6.70 |
| Sodium (mmol/L) | 150 (9.99) | 148 [130, 184] | 153.59 | 139.76 – 167.79 |
| Chloride (mmol/L) | 120 (9.01) | 120 [91.0, 151] | 120.86 | 108.29 – 134.26 |
| Phosphorus (mg/dL) | 5.72 (1.53) | 5.60 [1.60, 12.3] | 6.1 | 3.15 – 12.12 |
| Total Proteins (g/dL) | 4.83 (2.80) | 4.60 [1.10, 56.0] | 5.47 | 3.56 – 7.44 |
| Triglycerides (mg/dL) | 110 (150) | 58.5 [5.00, 1420] | 73.45 | 6.09 – 809.21 |
| A/G ratio | 0.281 (0.0510) | 0.280 [0.160, 0.430] | 0.15 | 0.10 – 0.24 |
| Albumin (%) | 21.6 (3.09) | 21.6 [13.7, 29.3] | 13.32 | 8.82 – 19.68 |
| Alpha 1 Globulins (%) | 1.45 (0.857) | 1.40 [0, 5.40] | 2.38 | 1.29 – 4.27 |
| Alpha 1 Globulins (g/dL) | 0.0658 (0.0400) | 0.0600 [0, 0.280] | 0.13 | 0.05 – 0.22 |
| Alpha 2 Globulins (%) | 24.5 (3.70) | 24.2 [13.8, 41.1] | 14.81 | 10.45 – 20.89 |
| Alpha 2 Globulins (g/dL) | 1.19 (0.659) | 1.13 [0.240, 12.2] | 0.8 | 0.48 – 1.36 |
| Beta Globulins (%) | 42.3 (4.58) | 42.3 [25.1, 63.1] | 47.66 | 35.41 – 59.54 |
| Beta Globulins (g/dL) | 2.05 (1.37) | 1.96 [0.450, 27.4] | 2.58 | 1.48 – 3.66 |
| Gamma Globulins (%) | 9.92 (3.24) | 9.60 [3.70, 22.6] | 20.53 | 14.02 – 29.73 |
| Gamma Globulins (g/dL) | 0.477 (0.282) | 0.431 [0.0900, 4.31] | 1.12 | 0.51 – 1.72 |
| Corticosterone (ng/mL) | 15.3 (15.5) | 9.63 [0.300, 98.6] | 2.85 | 0.65 – 14.64 |
| Hydroxybutyrate (mmol/L) | 0.824 (0.616) | 0.690 [0.0700, 5.88] | 0.18 | 0.05 – 0.64 |
| Bile acid | 11.5 (11.6) | 8.30 [0, 102] |  |  |
